# Endoplasmic reticulum–mitochondria contact sites are signalling hubs connecting nutrient sensing and GLP-1 secretion in L cells of the mouse gut: from physiology to obesity and type 2 diabetes

**DOI:** 10.1007/s00125-026-06693-7

**Published:** 2026-03-08

**Authors:** Alexandre Humbert, Margaux Nawrot, Nadia Bendridi, Yves Gouriou, Nicolas Bertocchini, Marie-Agnès Chauvin, Jingwei Ji-Cao, Christine Durand, Aurélie Vieille-Marchiset, Claudie Pinteur, Béatrice Morio, Frank Reimann, Bruno Guerci, Magalie A. Ravier, Sophie Lestavel, Jennifer Rieusset

**Affiliations:** 1 Université Claude Bernard Lyon 1, CarMeN Laboratory, Inserm U1060, INRAE U1397, Pierre-Bénite, France; 2 Institute of Metabolic Science, Addenbrooke’s Hospital, Cambridge, UK; 3 Department of Endocrinology, Diabetology, and Nutrition, Brabois Adult Hospital and University of Lorraine, Vandoeuvre-lès-Nancy, France; 4 IGF, University of Montpellier, CNRS, Inserm, Montpellier, France; 5 Université de Lille, Inserm, CHU Lille, Institut Pasteur de Lille, U1011-EGID, Lille, France

**Keywords:** Enteroendocrine cells, GLP-1, Glucose homeostasis, Mitochondria-associated membrane, Nutrient sensing, Obesity, Type 2 diabetes

## Abstract

**Aims/hypothesis:**

Postprandial glucagon-like peptide-1 (GLP-1) secretion by enteroendocrine L cells of the gut plays an important role in glucose homeostasis, thus representing a therapeutic option of ever-growing significance for type 2 diabetes. However, the precise mechanisms linking nutrient sensing and GLP-1 secretion are incompletely understood. In this study, we focused on a potential new role for endoplasmic reticulum (ER)–mitochondria contact sites, called mitochondria-associated membranes (MAMs), in nutrient-induced GLP-1 secretion by L cells, as they are dynamically regulated by nutrients, they influence cellular calcium homeostasis crucial for hormone secretion, and their miscommunication has been implicated in alterations of glucose homeostasis in several tissues.

**Methods:**

We combined biochemical and imaging approaches to investigate nutrient-induced GLP-1 secretion, and ER–mitochondria interaction and calcium exchange in the STC-1 cell line, ex vivo ileal mouse organoids, and/or in vivo in gut enteroendocrine cells from Glu-Venus mice, both in acute conditions and after diet-induced obesity and type 2 diabetes.

**Results:**

We show here that ER–mitochondria interactions are dynamically induced by two GLP-1 secretagogues, glucose and deoxycholic acid (DCA), in STC-1 cells (1.8- and 2.1-fold, respectively), ileal mouse organoids (1.7- and 1.3-fold, respectively), and in vivo in colonic L cells of Glu-Venus mice (1.3- and 1.2-fold, respectively). In addition, glucose increased ER–mitochondria calcium exchange in STC-1 cells (1.2-fold). A paracrine action of secreted GLP-1 was also involved in the regulation of MAMs by glucose and DCA in STC-1 cells. Dynamic reinforcement of MAMs by glucose and DCA played a causal role in GLP-1 release, as both pharmacological and genetic disruption of organelle communication blocked L cell secretory response to the two stimuli in STC-1 cells. In agreement, depleting ER calcium levels or inhibiting mitochondrial calcium entry decreased glucose-induced GLP-1 secretion (−37.5% and −30.9%, respectively), whereas inducing ER or mitochondrial stress prevented it (−47.9% and −51.8%, respectively). Mechanistically, glucose induces ER–mitochondria communication through a sodium–glucose cotransporter 1-mediated electrogenic effect, whereas DCA acts through a Takeda G protein-coupled receptor 5 (TGR5)–cAMP–protein kinase A (PKA) pathway. Finally, we demonstrated in C57Bl/6J mice and in Glu-Venus mice that diet-induced obesity reinforced basal ER–mitochondria interactions in colonic L cells and blocked their ability to respond to oral glucose in terms of both GLP-1 secretion and MAM upregulation.

**Conclusions/interpretation:**

These results point to a new role for ER–mitochondria calcium coupling in glucose-induced GLP-1 secretion in L cells of the gut, which is impaired in obesity and type 2 diabetes, providing a novel target for the modulation of GLP-1 secretion. Therefore, these data reinforce the potential targeting of MAMs to improve glycaemic outcomes in metabolic diseases.

**Graphical Abstract:**

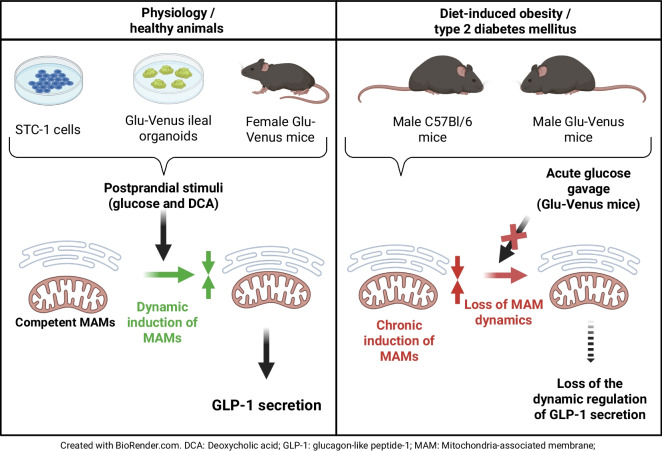

**Supplementary Information:**

The online version of this article (10.1007/s00125-026-06693-7) contains peer-reviewed but unedited supplementary material.


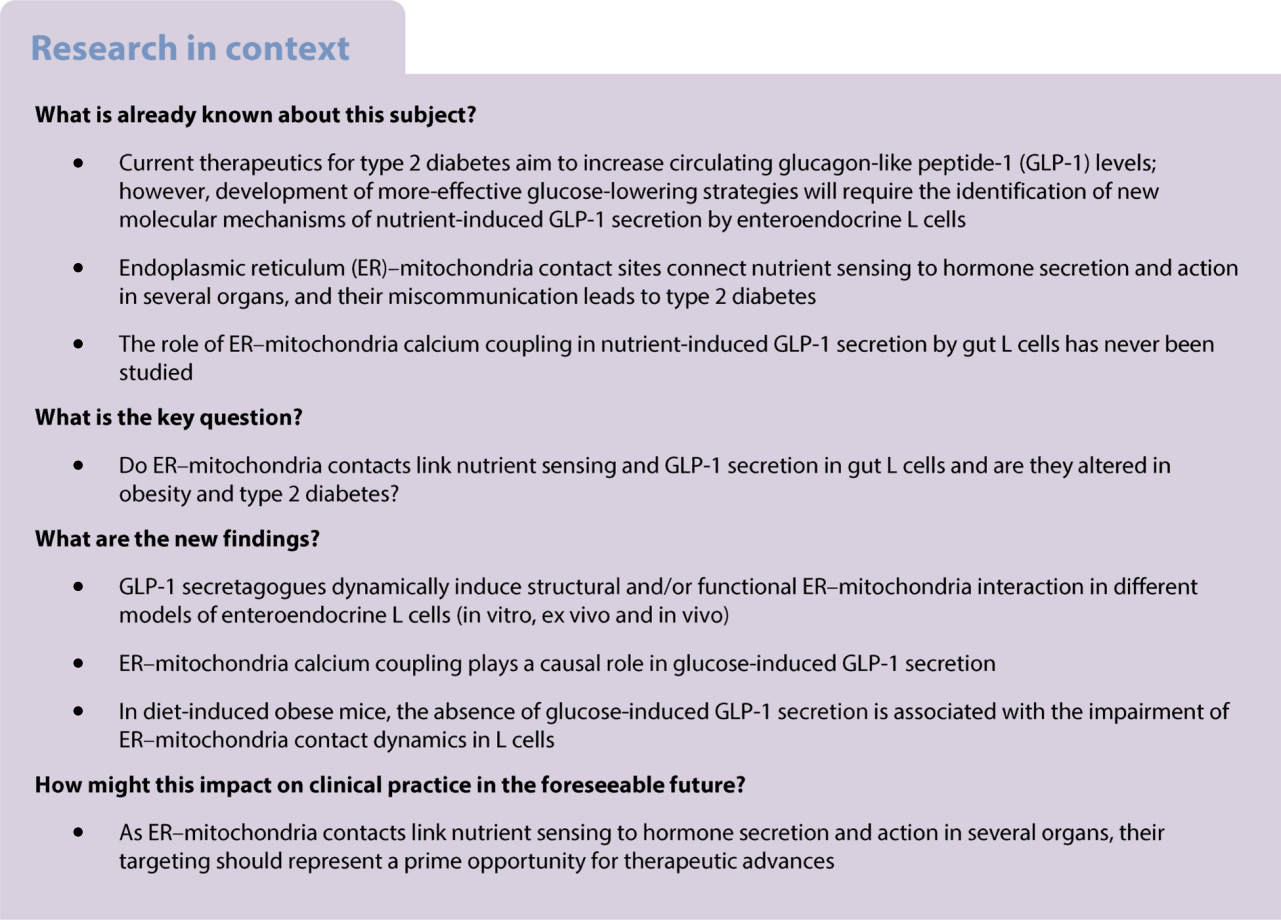


## Introduction

The gut hormone glucagon-like peptide-1 (GLP-1), is a key hormone regulating glucose levels and satiety in humans [1]. Secreted by enteroendocrine L cells after nutrient ingestion [2], its release is stimulated by glucose, lipids, proteins and non-nutrient stimuli, such as bile acids [2]. GLP-1 is a major incretin, enhancing insulin secretion via GLP-1 receptors (GLP-1Rs) on pancreatic beta cells (50–70% of postprandial insulin) [3] and through neuronal pathways [4], thereby lowering postprandial glucose levels. In type 2 diabetes, nutrient-induced GLP-1 secretion is impaired, contributing to hyperglycaemia [5]. Because of its role in glucose homeostasis, the GLP-1 pathway is a therapeutic target for type 2 diabetes and obesity. Current treatments use GLP-1R agonists (GLP-1RAs) or dipeptidyl peptidase-4 (DPP4) inhibitors to increase GLP-1 levels [6]. GLP-1RAs also reduce body weight [6] but their subcutaneous administration and digestive side effects limit compliance [7]. Thus, new mechanisms linking nutrient sensing to GLP-1 secretion must be identified to improve glucose-lowering strategies.

Mitochondria–endoplasmic reticulum (ER) contact (MERC) sites, known as mitochondria-associated membranes (MAMs), have emerged as key regulators of glucose homeostasis. ER–mitochondria miscommunication is linked to type 2 diabetes in several tissues, making MAMs a potential therapeutic target [8]. MAMs mediate phospholipid and calcium exchange, thereby controlling mitochondrial bioenergetics and cellular homeostasis [9]. Our pioneering work over the past decade identified MAMs as hubs regulating insulin action in liver [10] and skeletal muscle [11], where miscommunication is associated with insulin resistance [10, 11], in both mouse models and human cells. However, MAM reinforcement was also observed in obesity in liver [12] and skeletal muscle [13], suggesting a dual role. Importantly, we showed that ER–mitochondria miscommunication is an early causal event in hepatic and muscular insulin resistance, also seen in individuals with type 2 diabetes [14], although its molecular mechanisms remain unknown. We further uncovered a role of MAMs in pancreatic beta cells [15], where dysfunction occurs under glucotoxicity [15] and in type 2 diabetes [16], impairing glucose-stimulated insulin secretion. Last, MAMs are dynamically regulated by glucose levels in the liver, in order to adapt hepatic metabolism to glucose availability [17]. Altogether, these findings highlight MAMs as central hubs linking nutrient sensing to hormone secretion and action. Since the intestine is also crucial for glucose homeostasis, and GLP-1 secretion depends on calcium homeostasis [2], the role of MAMs in nutrient-induced GLP-1 secretion in intestinal L cells remains unexplored and therefore requires investigation.

Here, we investigated for the first time the potential role of MAMs in nutrient-induced GLP-1 secretion and their alterations in nutrient-induced obesity and type 2 diabetes, using dynamic imaging and functional approaches in murine enteroendocrine cell lines, L cells from ex vivo mouse ileal organoids and in vivo intestinal tissue from Glu-Venus mice. This last model uniquely allows analysis of MAMs in fluorescent enteroendocrine L cells, representing only 1–2% of the gut epithelium.

## Methods

### Cell culture

STC-1 cells (ATCC) were cultured on collagen-coated dishes (passage 17–30), in RPMI 1640 medium (Gibco, 31870-025) supplemented with 10% (vol./vol.) FBS (Sigma-Aldrich, F7524), 1% (vol./vol.) penicillin/streptomycin/amphotericin B (Gibco, 15240-062) and 1% (vol./vol.) GlutaMAX (Gibco, 35050-038), at 37°C and 5% (vol./vol.) CO_2_. GLUTag cells, graciously donated by D. J. Drucker, University of Toronto, were cultured in 90% (vol./vol.) complete medium (DMEM 1 g/l glucose [Gibco, 21885-025]) supplemented with 10% (vol./vol) FBS, 4 mmol/l glutamine, 1% (vol./vol.) penicillin/streptomycin/amphotericin B and 10% (vol./vol.) conditioned medium (complete medium that has already been brought into contact with cells and then filtered at 2 µm) at 37°C and under 5% (vol./vol.) CO_2_. For treatments, see electronic supplementary material (ESM) Methods for details.

### Ileum-derived mouse organoids

Ileal organoids were derived from a 5-week-old Glu-Venus female mouse, following StemCell Technologies’ protocol (see ESM Methods for details).

### GLP-1 secretion

GLP-1 secretion was assessed using an ELISA, targeted against active GLP-1 (7–37), according to the manufacturer’s protocol (EGLP-35K from Millipore) (see ESM Methods for details).

### In situ proximity ligation assay

ER–mitochondria interactions were assessed using an in situ proximity ligation assay (PLA, Duolink kit from Sigma) targeting either reticular inositol 1,4,5-triphosphate receptor (IP3R) 1 (Merck, 07-1210) and mitochondrial voltage-dependent anion channel 1 (VDAC1; Abcam, ab14734), or vesicle-associated membrane protein-associated protein B (VAPB; Proteintech, 66191) and protein tyrosine phosphatase interacting protein 51* (*PTPIP51; Proteintech, 20641), as described previously [18] (see ESM Methods for details).

### Transmission electron microscopy

Analysis of ER–mitochondria interaction on STC-1 cells was performed by transmission electron microscopy (TEM), as described previously [15, 17] (see ESM Methods for details).

### Calcium imaging

Calcium measurements were performed at 37°C using organelle-specific calcium probes and a wide-field Leica DMI6000B microscope equipped with a ×40 objective and an ORCA-Flash4.0 digital camera (HAMAMATSU) (see ESM Methods for details).

### Mitochondrial oxygen consumption

Oxygen consumption rate was measured into intact STC-1 cells using a Seahorse XFe 24 metabolic flux analyser (Agilent Technologies) (see ESM Methods for details).

### Mouse experiments

Animal studies in either C57Bl/6JOlaHsd male mice (Envigo, France, 057) or Glu-Venus female and male mice (B6 background) [19] were performed in accordance with the French guidelines for the Care and Use of Laboratory Animals and approved by the regional ethic committee (APAFIS 39264-202208301526991) (see ESM Methods for details).

### Statistical analysis

Statistical analyses were performed using GraphPad 8 and data are expressed as the mean ± SEM (see ESM Methods for details).

## Results

### Nutrient-induced GLP-1 secretion is associated with increased ER–mitochondria interactions and calcium exchange

**Fig. 1:**
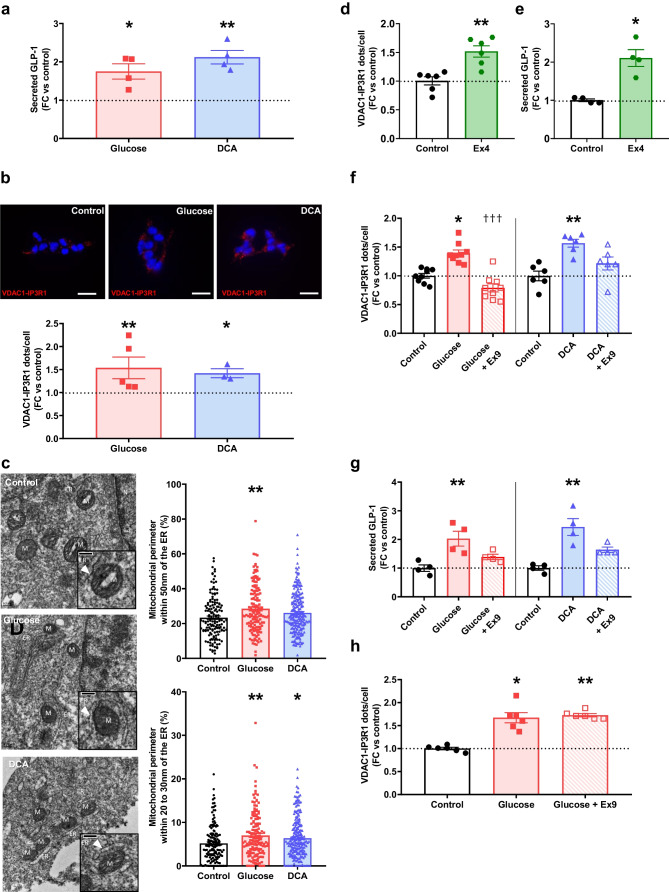
ER–mitochondria interactions are induced by GLP-1 secretagogues in STC-1 cells. (**a**) Effect of glucose (5 mmol/l) and DCA (30 µmol/l) treatments for 1 h on GLP-1 secretion assayed by ELISA. *n*=4 experiments in triplicate. *p<0.05, **p<0.01 (Kruskal–Wallis test followed by Dunn’s multiple comparisons). (**b**) Effect of glucose and DCA treatments for 1 h on ER–mitochondria interaction assessed by in situ PLA. Representative PLA images (scale bar, 25 µm) and quantification of VDAC1–IP3R1 proximity are shown. *n*=3–5 experiments in triplicate. *p<0.05, **p<0.01 (Kruskal–Wallis test followed by Dunn’s multiple comparisons). (**a**, **b**) The horizontal dotted line indicates the level of control where the treated conditions did not share the same controls. (**c**) Effect of glucose and DCA on total (<50 nm) and 20–30 nm ER–mitochondria contacts measured by TEM. Representative TEM images of mitochondria in contact with ER (scale bar, 0.2 μm) and quantifications are shown. For total contacts, *n*=147–218 mitochondria; for 0–30 nm, *n*=132–203 mitochondria, in one triplicate experiment. *p<0.05, **p<0.01 vs control (Kruskal–Wallis test followed by Dunn’s multiple comparison test). (**d**, **e**) Effects of Ex4 (100 nmol/l) for 1 h on MAMs (**d**) and GLP-1 secretion (**e**). *n*=4–6 in *n*=2 experiments. *p<0.05, **p<0.01 (Mann–Whitney test). (**f**, **g**) Effects of glucose for 1 h on MAMs (**f**) and GLP-1 secretion (**g**) in absence or presence of Ex9 (100 nmol/l). *n*=4–9 in *n*=2 or 3 experiments. *p<0.05, **p<0.01 vs control, ^†††^*p*<0.001 vs glucose (Kruskal–Wallis test followed by Dunn’s multiple comparison test). (**h**) Effect of glucose for 15 min in absence or presence of Ex9. *n*=6 in *n*=2 experiments. *p<0.05, **p<0.01 (Kruskal–Wallis test followed by Dunn’s multiple comparison test). FC, fold change; M, mitochondrion

To assess the role of MAMs in GLP-1 secretion, we stimulated STC-1 cells for 1 h with two different GLP-1 secretagogues for evaluating GLP-1 secretion by ELISA and ER–mitochondria interactions by in situ PLA. Both glucose (5 mmol/l) and deoxycholic acid (DCA, 30 µmol/l) significantly induced GLP-1 secretion after 1 h (Fig. 1a). The effect of glucose was associated with a significant reduction of intracellular GLP-1 content, whereas the effect of DCA was not (ESM Fig. 1a). Glucose and DCA concomitantly increased MAMs, as observed by the significant increase of VDAC1-IP3R1 dots per cell (Fig. 1b, 1.8- and 2.1-fold, respectively, and ESM Fig. 2a). To reinforce these data, we first confirmed that MAM regulation by glucose or DCA occurred as early as 15–30 min (ESM Fig. 2b), which is consistent with rapid GLP-1 secretion (ESM Fig. 2c). Second, we demonstrated by in situ PLA the effects of glucose and DCA on MAMs by targeting the VAPB-PTPI51 proximity, another protein tether of MAMs [20] (ESM Fig. 2d). Third, we used TEM to analyse the proportion of mitochondrial membranes in close contact (<50 nm) with ER, as well as the occurrence of contacts depending on the gap width. Glucose significantly increased total ER–mitochondria contacts, as well as calcium transfer-compatible 20–30 nm contacts, whereas DCA only significantly increased the 20–30 nm contacts (Fig. 1c). Last, glucose effects on MAMs and GLP-1 secretion were confirmed in GLUTag cells (ESM Fig. 3a, b).

As GLP-1R is expressed in gut enteroendocrine cells [21], where their activation by secreted GLP-1 stimulates GLP-1 release [22], we investigated whether the paracrine action of GLP-1 contributed to the 1 h effects of glucose and DCA on MAMs. First, exendin 4 treatment ([Ex4], 100 nmol/l, a GLP-1RA) significantly increased MAMs (Fig. 1d) and GLP-1 secretion (Fig. 1e) in STC-1 cells. Exendin 9 ([Ex9], 100 nmol/l, a GLP-1R inhibitor) prevented the effect of glucose on MAMs but only partially prevented the effects of DCA (Fig. 1f), with concordant regulation of GLP-1 secretion (Fig. 1g). Nevertheless, Ex9 did not prevent the 15 min effect of glucose on MAMs (Fig. 1h), in favour with both direct and indirect effect of glucose on MAMs in a time-dependent manner.

**Fig. 2:**
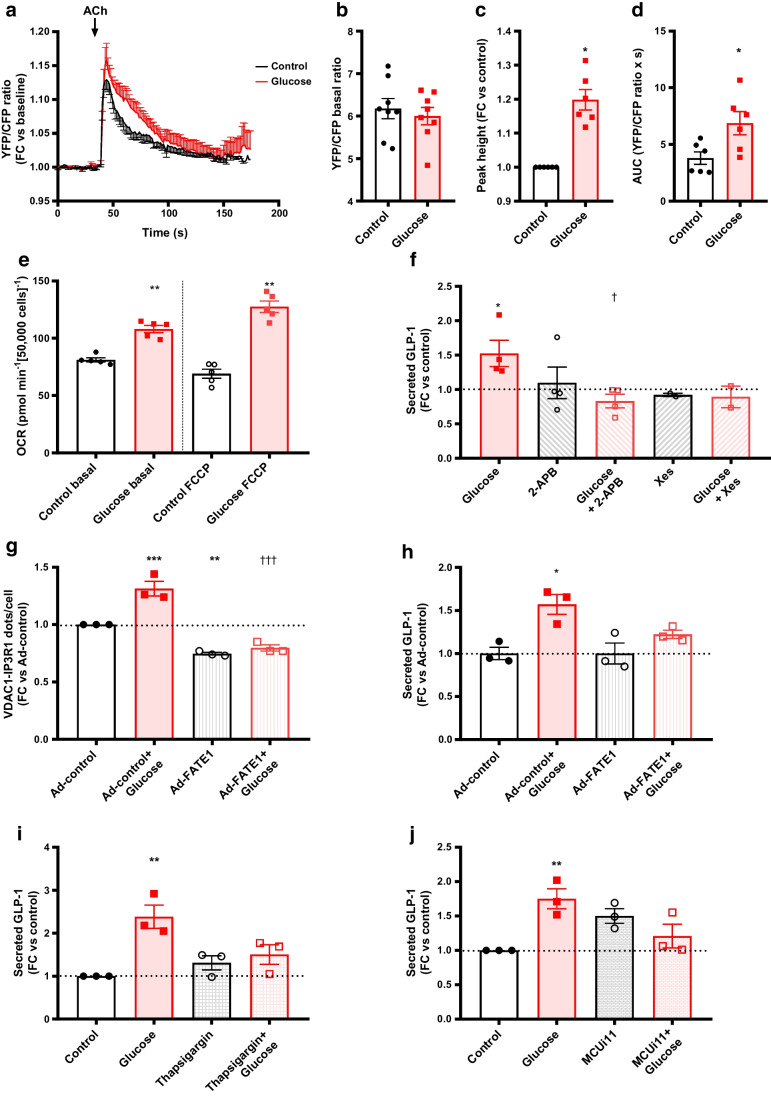
ER–mitochondrial calcium coupling is causally involved in glucose-induced GLP-1 secretion in STC-1 cells. (**a**–**d**) Mean curve of all six experimental days (**a**) and quantitative analysis of basal (**b**) and ACh-stimulated (**c**, **d**) mitochondrial calcium accumulation measured in STC-1 cells using the 4mtD3CPC probe, in the absence of extracellular calcium. Results for ACh stimulation are expressed as calcium peak amplitude after stimulation (**c**) and AUC (**d**). *n*=6–8 independent experiments, representing 62–66 cells. *p<0.05 (Mann–Whitney test). (**e**) Basal and FFCP-stimulated maximal mitochondrial oxygen consumption measured using a seahorse in basal or glucose-treated STC-1 cells upon 1 h. *n*=5. **p<0.01 (Mann–Whitney test). (**f**) Glucose-induced GLP-1 secretion is dampened by the inhibition of IP3R with 2-APB or Xes co-treatment; *n*=2–4 experiments in triplicate. The horizontal dotted line indicates the level of control where the treated conditions did not share the same controls. *p<0.05 for glucose effect vs control; ^†^*p*<0.05 for treatment effect vs glucose (one-way ANOVA followed by Holm–Sidak multiple comparisons test). (**g**, **h**) Glucose-stimulated MAM upregulation (**g**) and GLP-1 secretion (**h**) are lost after FATE1-mediated disruption of ER–mitochondria interaction. FATE1 was expressed using an adenovirus; *n*=3 independent experiments (**g**) or 3 triplicates in 1 experiment (**h**). *p<0.05, **p<0.01, ***p<0.001 for Ad-control + glucose or Ad-FATE1 effect vs Ad-control; ^†††^*p*<0.001 for Ad-FATE1 + glucose effect vs Ad-control (two-way ANOVA followed by Holm–Sidak multiple comparisons test). (**i**, **j**) Thapsigargin-induced ER stress (**i**) or MCUi11-mediated inhibition of the MCU (**j**) prevented glucose-induced GLP-1 secretion; *n*=3 independent experiments in triplicate. **p<0.01 (two-way ANOVA followed by Holm–Sidak multiple comparisons test). Ad, adenovirus; FC, fold change; OCR, oxygen consumption rate

We analysed the role of ER–mitochondria calcium coupling in GLP-1 secretion, a key MAM function [9]. First, we measured the acute effect of glucose and DCA on mitochondrial calcium levels, using the FRET-based 4mtD3CPV mitochondrial Ca^2+^ sensor [23], in the presence of extracellular calcium. Glucose acutely stimulated mitochondrial calcium accumulation in STC-1 cells (ESM Fig. 4a). As glucose stimulates extracellular calcium entry, both cytoplasm and ER can supply calcium to mitochondria. To test the involvement of reticular calcium, we measured the acute effect of glucose after the depletion of ER calcium stores by treatment with thapsigargin (1 µmol/l) for 10 min. Thapsigargin pre-treatment significantly reduced glucose-induced mitochondrial calcium accumulation without affecting basal calcium levels (ESM Fig. 4a). Similar effects were observed for DCA (ESM Fig. 4b). To specifically access ER–mitochondria calcium transfer, we imaged mitochondrial calcium accumulation in a calcium-free buffer and stimulated IP3R-mediated calcium release with different IP3R activators (ATP, histamine, acetylcholine [ACh]). Only ACh significantly increased mitochondrial calcium levels in our STC-1 clone (data not shown) and stimulated GLP-1 secretion in STC-1 cells (ESM Fig. 5). Importantly, 1 h glucose pre-treatment significantly enhanced ACh-mediated mitochondrial calcium accumulation, when compared with control cells without pre-treatment (Fig. 2a), as shown by the increase of both delta peak (Fig. 2c, 1.2-fold) and AUC (Fig. 2d). Basal mitochondrial calcium level was not modified (Fig. 2b). In agreement with mitochondrial calcium effect on mitochondria bioenergetics [24], glucose treatment stimulated both basal and FCCP-induced maximal mitochondrial oxygen consumption (Fig. 2e).

### Causal role of MAM regulation in nutrient-induced GLP-1 secretion

To validate the causal role of MAM regulation in glucose-induced GLP-1 secretion, we first used two different IP3R inhibitors (2-aminoethyl diphenyl borate [2-APB] and xestospongin C [Xes]) known to reduce IP3R-induced reticular calcium release [25, 26]. Glucose significantly induced GLP-1 secretion in untreated cells, whereas glucose-induced GLP-1 release was blunted in 2-APB- or Xes C-co-treated cells (Fig. 2f). A similar effect of 2-APB was found for DCA-induced GLP-1 secretion (ESM Fig. 6a). Although unspecific effects cannot be ruled out [25, 26], these results suggests that IP3R-mediated ER–mitochondria calcium exchange may regulate glucose-induced GLP-1 secretion.

We then experimentally disrupted MAMs in STC-1 cells by expressing the organelle spacer called fetal and adult testis expressed 1 (FATE1) [27] using an adenovirus (Ad-FATE1, 36 h) [11, 14]. FATE1 expression reduced VDAC1-IP3R1 proximity and prevented glucose-induced MAM flexibility (Fig. 2g). Importantly, FATE1-mediated MAM disruption prevented GLP-1 secretion induced either by glucose (Fig. 2h) or DCA (ESM Fig. 6b), validating the causal role of MAMs in nutrient-induced GLP-1 secretion. GLP-1 content in STC-1 cells was not modified by the chronic modulation of MAMs (ESM Fig. 1b).

To strengthen the findings on the role of reticular and mitochondrial calcium on GLP-1 secretion, we depleted each intracellular calcium compartment before assessing glucose-induced GLP-1 release. Thapsigargin treatment (2 µmol/l) depleted reticular calcium levels in 10 min, without modifying basal mitochondrial calcium levels (ESM Fig. 4c), and significantly reduced glucose-induced GLP-1 secretion (Fig. 2i, −37.5%). Similarly, MCUi11 treatment (20 µmol/l, inhibitor of the mitochondrial uniporter [MCU]) [28] significantly reduced both mitochondrial calcium entrance (ESM Fig. 4d) and KCl-induced GLP-1 secretion (Fig. 2j, −30.9%).

Last, to reinforce the role of ER and mitochondria homeostasis in GLP-1 secretion, we stressed the ER and mitochondria by using thapsigargin (0.3 µmol/l, 6 h, as performed previously [29]) and oligomycin (0.5 µmol/l, 1 h) treatments, respectively. Thapsigargin treatment reduced MAMs in basal condition and prevented the effect of glucose on MAMs (ESM Fig. 7a). Importantly, thapsigargin pre-treatment completely inhibited glucose-induced GLP-1 secretion (ESM Fig. 7b, −47.9%), whereas thapsigargin surprisingly increased basal GLP-1 secretion (ESM Fig. 7b). Similarly, oligomycin pre-treatment prevented the effect of glucose on MAMs (ESM Fig. 7c), and inhibited glucose-induced GLP-1 secretion (ESM Fig. 7d, −51.8%), without an effect on basal GLP-1 secretion (ESM Fig. 7d).

### Glucose regulates MAMs through an SGLT1-mediated electrogenic effect

**Fig. 3:**
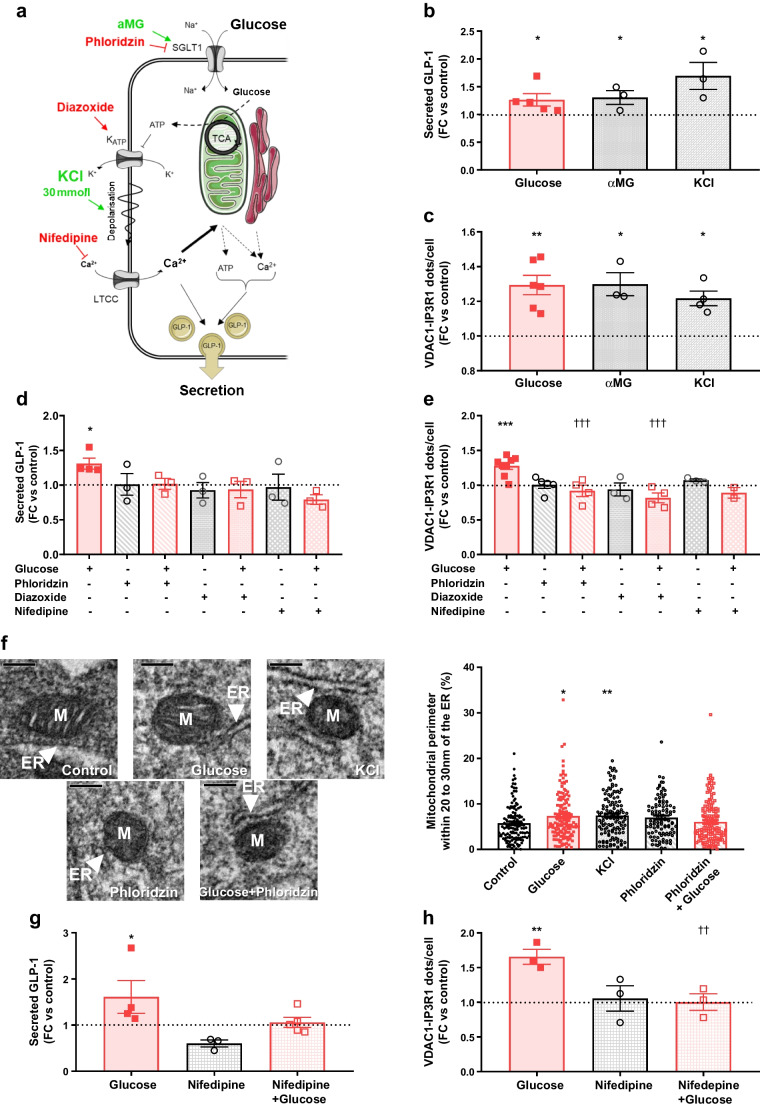
Glucose stimulates ER–mitochondria interaction through a SGLT1-mediated electrogenic effect. (**a**) Mechanisms of glucose action in L cells and sites of action of pharmacological compounds used. Agonists of the signalling pathways are shown in green, and antagonists are shown in red; dashed lines/arrows indicate that the involvement of a pathway is not clearly demonstrated in this study. Created with Servier Medical Art. (**b**) GLP-1 secretion following agonist stimulation of STC-1 cells. *n*=3–5 experiments in triplicate. *p<0.05 (Kruskal–Wallis test followed by Dunn’s multiple comparison test). (**c**) ER–mitochondria interaction assessed by in situ PLA in agonist-stimulated STC-1 cells. *n*=3–6 experiments in triplicate. *p<0.05, **p<0.01 (Kruskal–Wallis test followed by Dunn’s multiple comparison test). (**d**) GLP-1 secretion following antagonist treatment of STC-1 cells. *n*=3 or 4 experiments in triplicate. *p<0.05 (two-way ANOVA followed by Holm–Sidak multiple comparisons test). (**e**) ER–mitochondria interaction assessed by in situ PLA in antagonist-treated STC-1 cells. *n*=2–8 experiments in triplicate. ***p<0.01 for glucose effect vs control and ^†††^*p*<0.001 for treatment effect vs glucose (two-way ANOVA followed by Holm–Sidak multiple comparisons test). (**f**) Calcium transfer-compatible ER–mitochondria contacts (20–30 nm), showing representative TEM images of mitochondria in contact with ER (scale bar, 0.2 μm) and quantification. *n*=102–140 mitochondria in one triplicate experiment. *p<0.05, **p<0.01 vs control (Kruskal–Wallis test followed by Dunn’s multiple comparison test). (**g**) Effect of glucose on GLP-1 secretion in ileal organoids from Glu-Venus mice. *n*=3–5 experiments in duplicates. *p<0.05 (Kruskal–Wallis test followed by Dunn’s multiple comparisons). (**h**) Effect of glucose on ER–mitochondria interaction assessed by in situ PLA in Venus-positive L cells. *n*=3 experiments. **p<0.01 for glucose effect vs control; ^††^*p*<0.01 for nifedipine effect vs glucose (two-way ANOVA followed by Holm–Sidak multiple comparisons test). (**b**–**e**, **g**, **h**) The horizontal dotted line indicates the level of control where the treated conditions did not share the same controls. FC, fold change; LTCC, L-type voltage-dependent calcium channel; M, mitochondrion; TCA, tricarboxylic acid

Pathways regulating MAMs are only partially defined and may vary by tissue. We used pharmacological tools to explore mechanisms underlying glucose-mediated MAM upregulation (Fig. 3a). Glucose stimulates GLP-1 release through sodium–glucose cotransporter 1 (SGLT1)-mediated glucose entry, the membrane depolarisation through the closure of ATP-dependent K^+^ (K_ATP_) channels, and the activation of voltage-gated calcium channels (VDCCs) [30, 31]. As an agonist of this pathway, methyl-α-d-glucopyranoside (αMG, 5 mmol/l, a non-metabolised analogue of glucose) induced concomitant GLP-1 secretion (Fig. 3b) and MAMs (Fig. 3c). Similarly, KCl treatment (30 mmol/l, induces membrane depolarisation) reinforced MAMs (Fig. 3c) and induced GLP-1 release (Fig. 3b). As an antagonist of this pathway, phloridzin (1 mmol/l, inhibitor of SGLT1) concomitantly blocked glucose-stimulated GLP-1 release (Fig. 3d) and MAM upregulation (Fig. 3e). Furthermore, the forced opening of K_ATP_ channels by diazoxide (100 µmol/l) also prevented glucose-mediated regulation of GLP-1 release (Fig. 3d) and MAMs (Fig. 3e). Last, inhibition of VDCC by nifedipine (5 µmol/l) similarly inhibited glucose effects on GLP-1 release (Fig. 3d) and MAMs (Fig. 3e). Similar effects of nifedipine were also found in GLUTag cells (ESM Fig. 3a, b).

TEM analyses confirmed that both glucose and KCl increased MAMs, whereas phloridzin prevented the reinforcement of organelle contacts by glucose (Fig. 3f). To reconciliate glucose-induced electrogenic effects on MAMs with those of secreted GLP-1, we propose a potential crosstalk between GLP-1R signalling and this electrogenic pathway, as reported in pancreatic beta cells [32]. In agreement, diazoxide treatment prevented the effect of Ex4 on MAMs (ESM Fig. 8a) and GLP-1 secretion (ESM Fig. 8b), confirming the crosstalk between GLP-1R signalling and the glucose-stimulated electrogenic pathway in STC-1 cells.

As a more physiological model, we used ileum-derived organoids from a female Glu-Venus mouse; this mouse expresses Venus specifically in proglucagon-expressing cells, enabling MAM analysis in fluorescent L cells [19]. Venus-fluorescent L cells are present in ileum-derived organoids (ESM Fig. 9), and these cells responded to glucose, as shown by the induction of GLP-1 secretion (Fig. 3g). Furthermore, the glucose-induced GLP-1 secretion was prevented by nifedipine co-treatment (Fig. 3g), validating the electrogenic regulation of GLP-1 secretion by glucose in this model. Glucose treatment concomitantly induced MAMs, as observed by the increase of VDAC1-IP3R1 dots/fluorescent L cell (Fig. 3h, 1.7-fold), and nifedipine co-treatment prevented this effect (Fig. 3h). Therefore, glucose acutely reinforces MAMs via an SGLT1-mediated electrogenic effect in both STC-1 cells and ileal organoid L cells.

### DCA regulates MAMs through the Takeda G-protein-coupled receptor 5–cAMP–protein kinase A signalling pathway

**Fig. 4:**
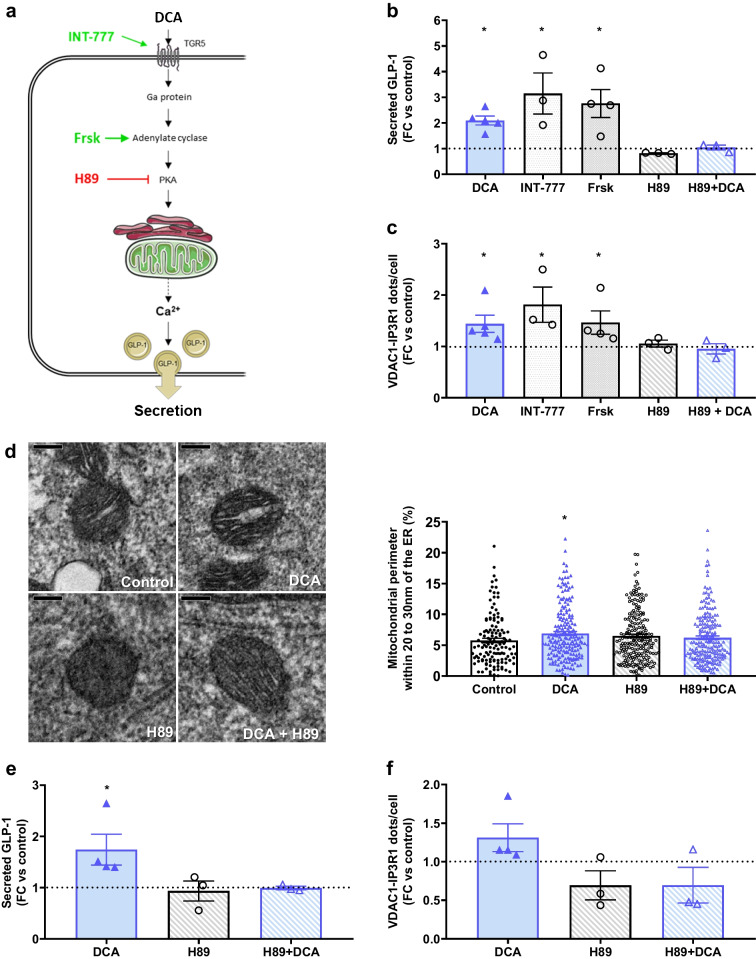
DCA stimulates ER–mitochondria interaction through the TGR5–cAMP–PKA pathway. (**a**) Mechanisms of bile acids’ action in L cells and sites of action of pharmacological compounds used. Agonists of the signalling pathway are shown in green, and antagonists are shown in red. Created with Servier Medical Art. (**b**) GLP-1 secretion following stimulation in STC-1 cells. *n*=3–5 experiments in 2 or 3 replicates. *p<0.05 (Kruskal–Wallis test followed by Dunn’s multiple comparison test). (**c**) ER–mitochondria interaction assessed by in situ PLA in STC-1 cells. *n*=3–5 experiments in triplicate. *p<0.05 (Kruskal–Wallis test followed by Dunn’s multiple comparison test). (**d**) Calcium-transfer-compatible ER–mitochondria contacts (20–30 nm) assessed by TEM. Representative images (scale bar, 0.2 µm) and quantification are shown. *n*=132–211 mitochondria. *p<0.05 vs control (Kruskal–Wallis test followed by Dunn’s multiple comparison test). (**e**, **f**) Effects of DCA on GLP-1 release (**e**) and ER–mitochondria interactions (**f**) in ileal organoids from Glu-Venus mice. *n*=3–4 experiments in duplicate. *p<0.05 (Kruskal–Wallis test followed by Dunn’s multiple comparison test). (**a**–**c**, **e**, **f**) The horizontal dotted line indicates the level of control where the treated conditions did not share the same controls. FC, fold change; Frsk, forskolin

Next, we applied a similar pharmacological approach to identify the mechanisms by which DCA upregulates MAMs in STC-1 cells (Fig. 4a). Bile acids are known to induce GLP-1 secretion through a Takeda G-protein-coupled receptor 5 (TGR5)–cAMP–protein kinase A (PKA) signalling pathway [33]. The TGR5 activator INT-777 concomitantly increased GLP-1 secretion (Fig. 4b) and MAMs (Fig. 4c). Similarly, the induction of cAMP formation by forskolin induced GLP-1 release (Fig. 4b) and reinforced MAMs (Fig. 4c). Last, the inhibition of PKA by H89 prevented DCA effects on GLP-1 release (Fig. 4b) and MAMs (Fig. 4c). We confirmed the regulation of MAMs by DCA with and without H89 treatment using TEM (Fig. 4d). Similar results were confirmed in ileum-derived organoids from Glu-Venus mice for both GLP1 secretion (Fig. 4e) and MAMs (Fig. 4f, 1.3-fold). Therefore, bile acids induce GLP-1 secretion by regulating MAMs via the TGR5–cAMP–PKA signalling pathway in both STC-1 cells and ileal organoid L cells.

### Nutrient-induced GLP-1 secretion is associated with increased ER–mitochondria interactions in L cells from mouse gut

**Fig. 5:**
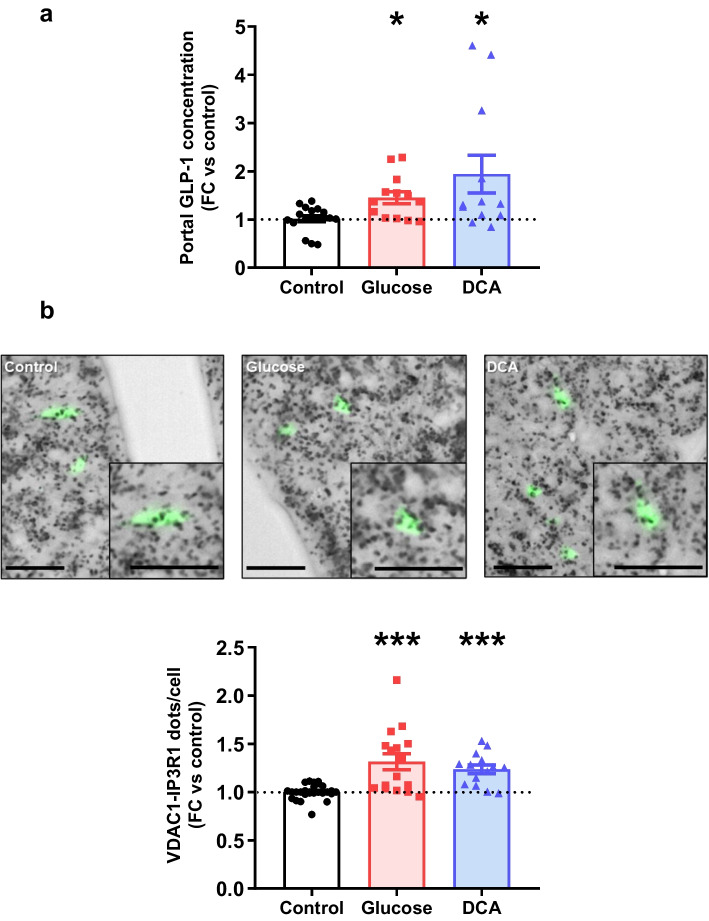
MAMs link glucose and DCA sensing to GLP-1 secretion in colonic L cells of Glu-Venus mice. Healthy female Glu-Venus mice were acutely (30 min) force-fed with glucose (0.2 mg/kg) or DCA (15 mg/kg). (**a**) Portal-vein GLP-1 levels measured by ELISA. *n*=12–19 mice/group. *p<0.05 (Kruskal–Wallis test followed by Dunn’s multiple comparison test). (**b**) ER–mitochondria interaction in Venus-positive colonic L cells. PLA images (scale bar, 25 µm; Venus-positive L cells are indicated in green; VDAC1-IP3R1 dots appear in black) and quantification are shown. *n*=14–27 mice/group. ***p<0.001 (Kruskal–Wallis test followed by Dunn’s multiple comparison test). FC, fold change

To assess the in vivo relevance of our findings, we investigated the acute effect (30 min) of glucose (0.2 mg/kg) or DCA (15 mg/kg) on portal-vein GLP-1 levels and on MAMs in colonic L cells from female Glu-Venus mice, as this segment has the highest density of GLP-1-expressing cells in multiple species [34]. Both glucose and DCA significantly increased portal-vein GLP-1 levels (Fig. 5a) and concomitantly increased MAMs, as evidenced by the increase in VDAC1-IP3R1 proximity in colonic Venus-positive L cells measured by in situ PLA (Fig. 5b, 1.3-fold and 1.2-fold, respectively). We also explored the regulation of MAMs in ileal Venus-positive L cells and confirmed their induction by glucose and DCA, although the effect reached the level of significance only with glucose (ESM Fig. 10), in agreement with the preferential site of action of glucose in the gut [2].

### Diet-induced obesity is associated with increased MAMs in mouse gut L cells

**Fig. 6:**
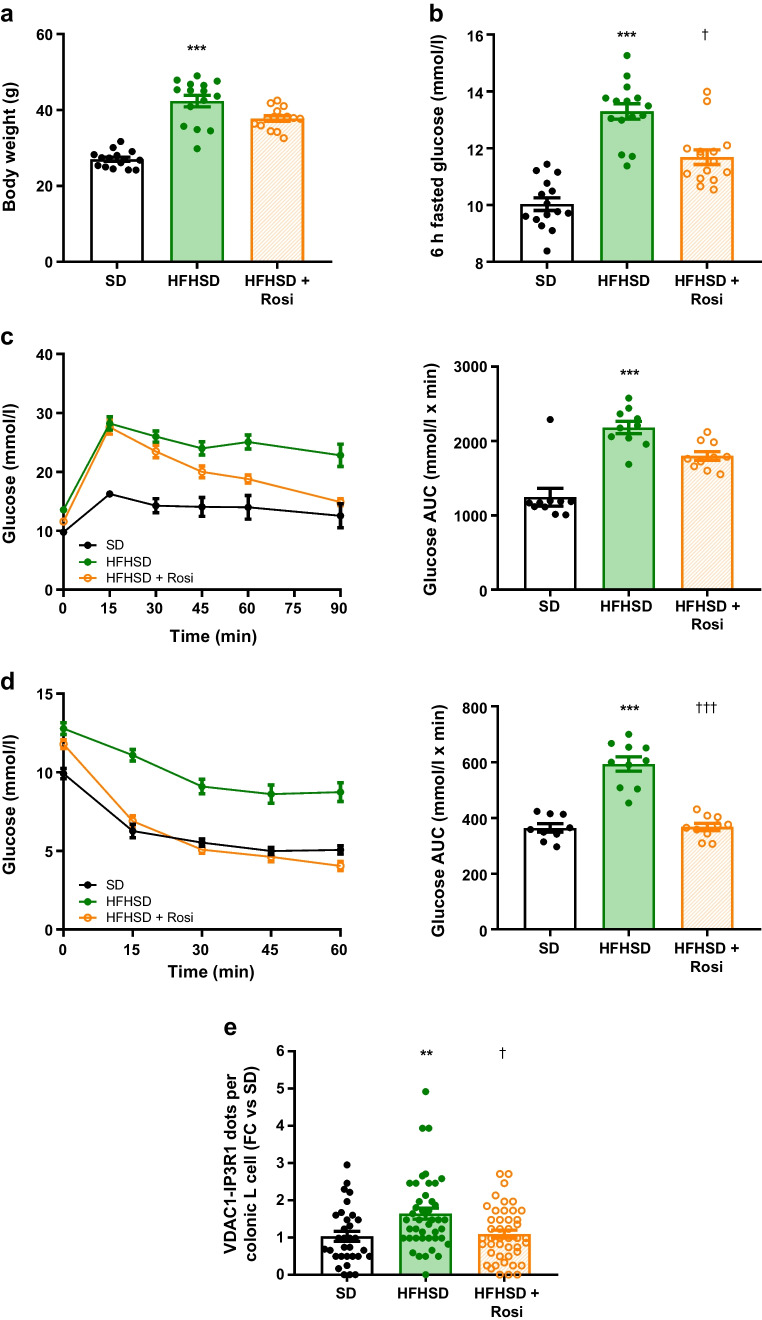
Reinforcement of MAMs in colonic L cells of diet-induced obese mice. C57Bl/6J male mice were fed with an SD or HFHSD for 16 weeks. A group of HFHSD-fed mice were orally treated with rosiglitazone (10 mg/kg) for the last 6 weeks of the nutritional protocol. (**a**–**d**) Metabolic phenotyping of mice: body weight (**a**); 6 h fasted glucose levels (**b**); and glucose levels and AUC for glucose in IPGTT (**c**) and ITT (**d**). *n*=9–15 mice/group. ***p<0.001 for HFHSD effect vs SD; ^†^*p*<0.05, ^†††^*p*<0.001 for rosiglitazone effect vs HFHSD (Kruskal–Wallis test followed by Dunn’s multiple comparison test). (**e**) ER–mitochondria interactions in GLP-1-positive colonic L cells in SD-, HFHSD- and HFHSD+Rosi-fed mice. *n*=32–44 pictures taken from *n*=3 or 4 mice/group. **p<0.01 for HFHSD effect vs SD; ^†^*p*<0.05 for rosiglitazone effect vs HFHSD (Kruskal–Wallis test followed by Dunn’s multiple comparison test). FC, fold change; Rosi, rosiglitazone

ER–mitochondria miscommunication has been linked to impaired insulin secretion and action in obesity/type 2 diabetes but whether MAMs are altered in gut L cells remains unknown. We therefore examined MAMs in colonic L cells of a nutritional model of obesity/type 2 diabetes. Male C57Bl/6J mice were fed a standard diet (SD) or a high-fat high-sucrose diet (HFHSD) for 16 weeks, with a subgroup receiving rosiglitazone (10 mg/kg) during the last 6 weeks (HFHSD+Rosi). HFHSD-fed mice developed obesity, hyperglycaemia, glucose intolerance and insulin resistance compared with SD-fed mice, and rosiglitazone treatment significantly improved hyperglycaemia and insulin sensitivity of HFHSD-fed mice (Fig. 6a–d). The glucose intolerance of HFHSD mice was partially improved by rosiglitazone (Fig. 6c), whereas the body weight was not modified (Fig. 6a). Importantly, HFHSD feeding increased MAMs in GLP-1-labelled L cells compared with SD feeding, whereas rosiglitazone treatment restored organelle contacts to a level close to that of the SD-fed mice (Fig. 6e).

### Loss of MAM regulation by glucose is associated with reduced GLP-1 secretion

**Fig. 7:**
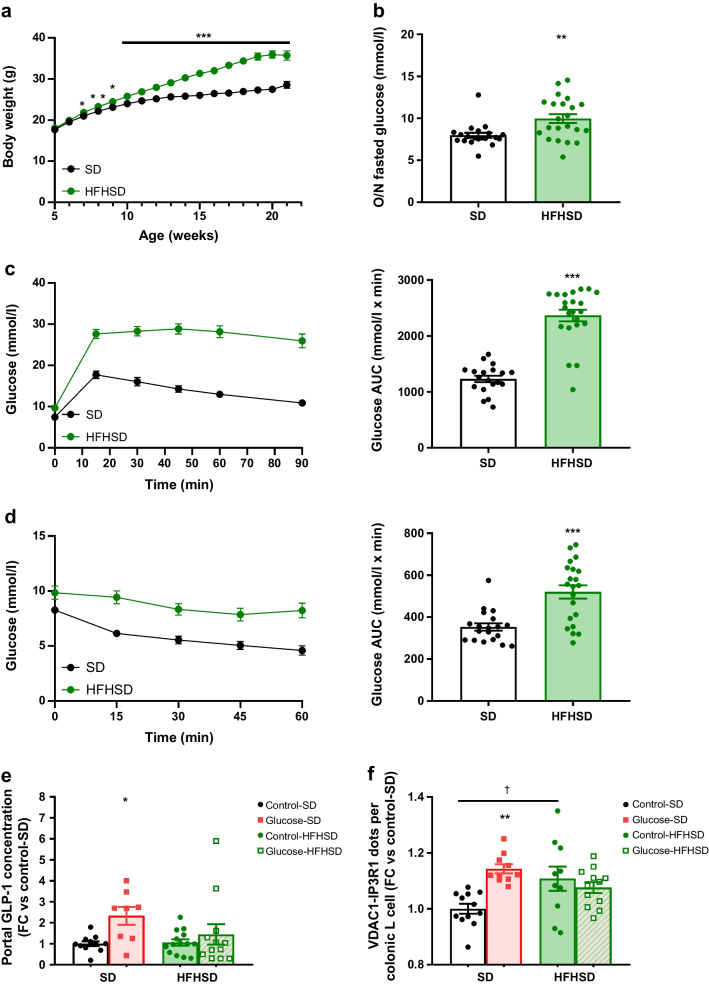
Diet-induced obesity impairs GLP-1 secretion, increases ER–mitochondria interaction and prevents glucose-induced MAM induction in Glu-Venus mice. Male Glu-Venus mice were fed with an SD or HFHSD for 16 weeks. After 6 h of fasting, mice were force-fed with glucose for 30 min. (**a**–**d**) Metabolic phenotyping of mice: body weight (**a**); overnight-fasted glucose levels (**b**); and glucose levels and AUC for glucose in IPGTT (**c**) and ITT (**d**). *n*=19–22 mice/group. **p<0.01, ***p<0.001 (multiple *t* tests [**a**] or Mann–Whitney test [**b**–**d**]). (**e**) Portal-vein GLP-1 concentration 30 min after an oral gavage in SD- or HFHSD-fed Glu-Venus mice. *n*=8–14 mice/group. *p<0.05 (two-way ANOVA followed by Sidak’s multiple comparisons test). (**f**) ER–mitochondria interaction in Venus-positive colonic L cells, 30 min after an oral gavage in SD- or HFHSD-fed Glu-Venus mice. *n*=10–12 mice/group. **p<0.01 for glucose-SD vs control-SD; ^†^*p*<0.05 for control-HFHSD vs control-SD group (two-way ANOVA followed by Sidak’s multiple comparisons test). FC, fold change; O/N, overnight

We next examined in vivo whether overnutrition-induced MAM reinforcement affects glucose-stimulated GLP-1 secretion by L cells. Male Glu-Venus mice were fed with SD or HFHSD for 16 weeks, before a 30 min glucose gavage. HFHSD-fed mice developed obesity, hyperglycaemia, glucose intolerance and insulin resistance compared with SD-fed mice (Fig. 7a–d). As expected, oral glucose administration increased portal-vein GLP-1 levels in SD-fed Glu-Venus mice, whereas this acute regulation was lost in HFHSD-fed mice (Fig. 7e), confirming altered glucose-stimulated GLP-1 secretion in obesity/type 2 diabetes [5, 35]. At the MAM level, we confirmed glucose-mediated increase of MAMs in colonic fluorescent L cells from Glu-Venus SD-fed mice, as well as the reinforcement of MAMs by HFHSD feeding (Fig. 7f). Importantly, glucose was not able to stimulate MAMs in L cells from Glu-Venus HFHSD mice (Fig. 7f), pointing to a loss of the nutritional regulation of MAMs in obesity/type 2 diabetes. Interestingly, these data were confirmed in vitro, as chronic high-glucose treatment of STC-1 cells (48 h) increased basal ER–mitochondria interactions and prevented glucose-stimulated MAM upregulation (ESM Fig. 11a) and GLP-1 secretion (ESM Fig. 11b).

## Discussion

MAMs are signalling hubs connecting nutrient sensing to hormone secretion/action to regulate glucose homeostasis in different metabolic tissues [8]. While it has been well studied for insulin, nothing is known about the gut hormone GLP-1 and whether MAMs could participate in nutrient-induced GLP-1 secretion by intestinal L cells. Here, we point out that acute upregulation of ER–mitochondria calcium coupling by glucose or DCA is required for GLP-1 release, through different signalling pathways in intestinal L cells. Furthermore, chronic reinforcement of MAMs and loss of their acute regulation by glucose are associated with reduced GLP-1 secretion in obese mice.

One novel finding is that glucose and DCA acutely reinforced ER–mitochondria communication in intestinal L cells. Even though these effects are modest, they are supported by extensive approaches: (1) MAM regulation in L cells was confirmed in different models (STC-1 and GLUTag cell lines, intestinal ex vivo organoids and in vivo in mouse gut); (2) in vivo, MAM regulation was confirmed in two different mouse strains, C57Bl/6J and Glu-Venus mice, and in male and female mice; and (3) structural MAM regulation was confirmed by complementary techniques, in situ PLA targeting different tethers and TEM, and was functionally associated with increased organelle calcium exchange. Furthermore, the kinetic study supports the role of MAMs in GLP-1 release, as their regulation by nutrients occurs between 15–60 min, in agreement with the biphasic secretion of GLP-1 [36]. For convenience, most of our experiments were performed after 1 h of treatment. Interestingly, at this time, the paracrine activation of GLP-1R on L cells by the secreted GLP-1 contributes to the effects of glucose and DCA on MAMs, pointing towards GLP-1 as a new regulator of MAMs. Nevertheless, we demonstrate that glucose can regulate MAMs both directly and indirectly, via secreted GLP-1, in a time-dependent manner. Last, MAMs are probably not involved in basal GLP-1 secretion but rather involved in sustained nutrient-induced GLP-1 secretion in postprandial state for two reasons: (1) both in vitro MAM modulation by FATE1 expression and HFHSD-induced chronic increase of MAMs in colonic mouse L cells did not modulate basal GLP-1 secretion but rather prevented glucose-induced MAM dynamics; and (2) secreted GLP-1 following nutrient stimulation acts in a paracrine manner in L cells at 1 h and stimulates MAMs.

Besides a correlative association, the following independent data collectively point to a causal role for MAMs in nutrient-induced GLP-1 secretion: (1) MAMs and GLP-1 secretion were systematically and concomitantly regulated in our different models; (2) the inhibition of IP3R-mediated calcium release using two different IP3R inhibitors or the chronic disruption of MAMs by FATE1 expression prevented nutrient-induced GLP-1 release; (3) both thapsigargin-mediated ER calcium depletion and MCUi11-mediated inhibition of mitochondrial calcium entrance blunted glucose-induced GLP-1 secretion; (4) ER or mitochondrial stress affected glucose-induced GLP-1 secretion; and (5) altered GLP-1 secretion in obese mice or in 48 h high-glucose-treated STC-1 cells was associated with a loss of glucose-induced MAM flexibility. Unfortunately, the causal role of MAMs in GLP-1 secretion is challenging to confirm in vivo, as no strategy currently exists to pharmacologically or genetically modulate MAMs specifically in gut L cells. Similarly, it cannot be confirmed ex vivo, as no method for lasting culture of pure primary L cells has been established. The mechanisms by which MAMs control GLP-1 secretion are currently elusive and require further investigations. Beyond cytoplasmic calcium levels, our data suggest that both ER calcium release and mitochondrial calcium accumulation through MAMs are crucial for GLP-1 secretion. However, we cannot exclude the possibility that modulation of mitochondrial bioenergetics and ATP synthesis also participates, since K_ATP_ channel closure and phosphorylation, in addition to exocytosis, are ATP dependent [2]. In agreement, we demonstrate that glucose stimulates mitochondrial oxygen consumption and that the induction of ER and mitochondrial stress reduces glucose-induced GLP-1 secretion.

Mechanisms connecting nutrient sensing to MAM regulation remain unknown. While glucose reduced MAMs in the liver [17], glucose acutely induced MAMs to support insulin secretion in pancreatic beta cells [15], indicating a tissue-specific regulation of MAMs by glucose according to tissue metabolic function. Here, glucose also acutely reinforced MAMs in L cells to support GLP-1 secretion, pointing to a crucial role for MAMs in connecting glucose sensing to hormone secretion in the postprandial state. For the first time, we demonstrate that membrane depolarisation and calcium entrance through VDCC are a new mechanism of glucose-mediated MAM regulation. In agreement, we found using both PLA and TEM that 30 mmol/l KCl treatment upregulates MAMs, pointing to a regulation of organelle communication by plasma membrane currents. This is in line with data in neurons where MAMs are regulated by electric currents [37] or in chromaffin cells where ER and mitochondria stress impair catecholamines exocytosis [38]. Although L cells are electrically excitable cells, whether MAMs are regulated by action potentials requires further investigations. In addition, the factors connecting intracellular calcium increase to ER–mitochondria interactions are currently unknown and also require further investigations. Interestingly, the fact that secreted GLP-1 participates in the effect of glucose on MAMs suggests a crosstalk between GLP-1R signalling and this electrogenic pathway. Such potentiation has previously been reported in pancreatic beta cells [32]. Indeed, besides its direct effect on granule exocytosis (via activities of PKA and exchange proteins directly activated by cAMP 2 [EPAC2]), GLP-1 may confer glucose competence to some beta cells by inhibiting further K_ATP_ channels to induce, in the presence of glucose membrane depolarisation, generation of action potentials and calcium influx [32]. It is likely that the GLP-1R–cAMP–PKA pathway phosphorylates and inhibits the K_ATP_ channels [39] and/or L-type VDCCs [40], as supported by our data showing that the effects of Ex4 on MAMs and GLP-1 secretion are prevented by diazoxide. In addition, we identified a new regulation of MAMs by bile acids through the TGR5–cAMP–PKA pathway, in line with the induction of MAMs by cAMP agonists in Leydig cells [41]. Therefore, phosphorylation of MAM proteins may be a key control point of organelle interaction, as evidenced by the targeting of IP3R [42] or mitofusin-2 (MFN2) [43] by PKA.

Last, ER–mitochondria miscommunication was associated with altered GLP-1 release with obesity/type 2 diabetes. Indeed, we observed a reinforcement of ER–mitochondria interactions in colonic L cells of HFHSD-fed mice, in both C57Bl/6J and Glu-Venus mice. Importantly, this chronic increase of MAMs prevents their acute upregulation by glucose in colonic L cells of HFHSD-fed mice, an effect also confirmed in STC-1 cells exposed to glucotoxicity, strongly supporting the notion that loss of MAM flexibility by nutrients participates in the alteration of GLP-1 release in the context of obesity/type 2 diabetes. Interestingly, we previously reported the disruption of MAMs in liver and skeletal muscle of the same nutritional model [11, 14], while MAMs are reinforced in gut L cells in the present study, suggesting that ER–mitochondria miscommunication in obesity/type 2 diabetes is tissue dependent. Interestingly, intestinal L cells resemble pancreatic beta cells in regard to MAM flexibility and hormone secretion. Beyond sharing similar regulatory mechanisms of glucose-induced hormone secretion, MAM dynamics seem similar in both pancreatic beta cells and intestinal L cells, with an acute induction by glucose to support hormone secretion and a loss of glucose regulation during chronic hyperglycaemia, likely participating in altered hormone release [15, 30, 35].

In conclusion, we demonstrate a new role for MAMs in nutrient-induced GLP-1 secretion and their alteration in the context of obesity/type 2 diabetes. As MAMs are crucial for the control of insulin secretion and action, we propose that MAMs could play an integrated role in the control of glucose homeostasis in different tissues in the postprandial state. Therefore, targeting MAMs could be a new and more efficient strategy to improve glycaemic outcomes in obesity/type 2 diabetes.

## Supplementary Information

Below is the link to the electronic supplementary material.

ESM (PDF 2.58 MB)

## Data Availability

The data of the current study are available from the corresponding author on reasonable request.

## Abbreviations

2-APB: 2-Aminoethyl diphenyl borate
ACh: Acetylcholine
DCA: Deoxycholic acid
DPP4: Dipeptidyl peptidase-4
ER: Endoplasmic reticulum
Ex4: Exendin 4
Ex9: Exendin 9
FATE1: Fetal and adult testis expressed 1
GLP-1: Glucagon-like peptide-1
GLP-1R: GLP-1 receptor
GLP-1RA: GLP-1R agonist
HFHSD: High-fat high-sucrose diet
IP3R: Inositol 1,4,5-trisphosphate receptor
K_ATP_: ATP-dependent K^+^
MAM: Mitochondria-associated membrane
MCU: Mitochondrial uniporter
αMG: Methyl-α-d-glucopyranoside
PKA: Protein kinase A
PLA: Proximity ligation assay
PTPIP51: Protein tyrosine phosphatase interacting protein 51
Rosi: Rosiglitazone
SD: Standard diet
SGLT1: Sodium–glucose cotransporter 1
TEM: Transmission electron microscopy
TGR5: Takeda G-protein-coupled receptor 5
VAPB: Vesicle-associated membrane protein-associated protein B
VDAC1: Voltage-dependent anion channel 1
VDCC: Voltage-gated calcium channels
Xes: Xestospongin C
