# Supplementary material for: Endoplasmic reticulum–mitochondria contact sites are signalling hubs connecting nutrient sensing and GLP-1 secretion in L cells of the mouse gut: from physiology to obesity and type 2 diabetes
Source: Diabetologia. 2026 Mar 8;69(7):2044–60. doi: 10.1007/s00125-026-06693-7 (PMC13236814; doi:10.1007/s00125-026-06693-7)
Supplement: Supplementary file 1 — ESM (PDF 2.58 MB) [file 125_2026_6693_MOESM1_ESM.pdf]

Endoplasmic reticulum–mitochondria contact sites are signalling hubs connecting nutrient sensing and GLP-1 secretion in L cells of the mouse gut: from physiology to obesity and type 2 diabetes

Humbert A et al.

Electronic Supplementary Material

Electronic supplementary material contains supplementary methods, as well as 11 supplementary figures.

Supplementary methods

Cell culture

All cell lines used were free of mycoplasmas. Cells were seeded at least 36h before experiments. Masking was not carried out. Treatments were biphasic, composed of a 1h preincubation in saline HEPES buffer followed by a second 1h incubation in HEPES buffer with the indicated compound or vehicle. Unless otherwise stated, all compounds were purchased from Sigma-Aldrich (Darmstadt, Germany). Treatments used were glucose (5mM, G8644), deoxycholic acid (DCA, 30µM, D2510), methyl-α-D-glucopyranoside (αMG, 5mM, M9376), phloridzin (1mM, PHL80513), KCl (30mM, osmotically compensated by NaCl reduction in the buffer), diazoxide (100µM, D9035), nifedipine (5µM, N7634), INT-777 (30µM, MedChemExpress, HY-15677), H89 (10µM, B1427), forskolin (10µM, F6886), 2-aminoethyl diphenyl borate (2-APB, 50µM, 100065), Exendin 4 (Ex4, 100nM, Abcam, ab120214) and Exendin 9 (Ex9, 100nM, Abcam, ab1411101). All non-water-soluble compounds were diluted in dimethyl sulfoxide (DMSO), and final DMSO concentration was 0.1-0.5%. For PLA experiments, cells were fixed in 10% paraformaldehyde (PFA, Thermo Fisher Scientific, J19943.K2) and permeabilized with Triton X-100 (X100). For glucagon-like peptide-1 (GLP-1) Enzyme-Linked Immunosorbent Assay (ELISA) experimentations, all media were supplemented with 2% Dipeptidyl-peptidase-4 (DPP4) inhibitor mix (Millipore, P27487). For organelle spacer experiments, cells were infected with recombinant adenoviruses expressing mCherry (Ad-mCherry, as a control) or FATE1 (Ad-FATE1) for 48h prior treatments, as previously described[1]. FATE1 is a testis-specific cancer antigen protein, which was originally described to reduce ER-mitochondria interactions and calcium transfer in adrenocortical cells[2]. Mechanistically, FATE1 resides in a high molecular weight complex at MAM interface, and could directly interact with mitofilin[2]. Importantly, FATE1 is weakly or not at all expressed in non-tumour tissues, particularly in metabolic tissues. Therefore, we expressed it into STC1 cells to disrupt ER-mitochondria interactions. The expression of an exogenous protein avoids to disrupt MAMs by invalidating endogenous protein that can have other functions outside MAMs.

Ileum-derived mouse organoids

Briefly, a 5-week old Glu-Venus mouse[2] was euthanised by cervical dislocation, a 10cm long segment of the ileum was harvested and placed in cold PBS, then flushed with cold PBS,

opened and cut in 2mm^2 squares, crypts were separated by pipetting, digested using the manufacturer's solution Gentle Cell Dissociation Reagent (STEMCELL Technologies, 100-0485), and incubated at room temperature for 15 min on a rocking platform. Tissue fragments were resuspended in PBS supplemented with 0.1% BSA and mechanically dissociated by repeated pipetting. After gravity sedimentation, crypt-containing supernatants were collected, passed through a 70µm filter, and centrifuged at 290 × g for 5 min at 4 °C. The crypt pellet was washed once with advanced DMEM/F-12 (Gibco, 12634010) containing 15 mmol/l HEPES.

Approximately 500 crypts were embedded in Matrigel Matrix (Corning, 356231) and seeded in 48-well plates. Organoids were free of mycoplasmas. They were cultured in IntestiCult Organoid Growth Medium (Mouse) (STEMCELL Technologies, 06005), supplemented with 1% gentamicin (Gibco, 15710049) and 10 µM Y-27632 (Merck, Y0503-1MG). After a few passages, the structures stabilised. Organoids were seeded at 90 organoids per dome of matrigel 48 h prior to the experiments. Treatments were as previously described for STC-1 cells, but DCA concentration was lowered to 1µM following the results of a GLP-1 secretory dose-response. For PLA experiments, approximately 270 organoids per condition were fixed in 10% formaldehyde for 30 min. After several PBS rinses, the organoids were stained with 0.1% neutral red (Sigma-Aldrich, N7005), then suspended in Histogel (Fisher Scientific, HG-4000-012). After 10 min of polymerization on ice, the organoids were dehydrated through a graded ethanol series and subsequently embedded in paraffin. The samples were sectioned at 4 µm thickness using a microtome and mounted onto slides. For GLP-1 ELISA experiments, after the supernatant was retrieved, the organoids were resuspended in ice cold PBS supplemented by 2% DPP4 inhibitor mix. Masking was not carried out.

GLP-1 secretion

Active GLP-1 from cell medium or periportal mouse blood (both in presence of 2% DPP-4 inhibitor) was quantified by ELISA, following the recommendations of the manufacturer (EGLP-35K from Millipore). This ELISA was tested by Millipore for Ex4 cross-reactivity and no cross- reactivity was observed. In addition, there is very little sequence homology (53%) between Ex4 and native GLP-1. Only the first nine NH2-terminal amino acids are homologous (barring the second amino acid alanine in GLP-1, which has been replaced with glycine in Ex4). Results were calculated as the ratio of secreted GLP-1 divided by total (secreted + cellular) GLP-1, and expressed as a fold of activation compared to the control.

Plasma samples were dosed directly, cellular supernatant was diluted 50 times, and cellular pellets were diluted 2500 times. Masking was not carried out.

In situ proximity ligation assay

In situ proximity ligation assay (PLA) was used to quantified ER-mitochondria proximity as organelle distance in MAMs is compatible with the 40nm distance required for proximity ligation. PLA was mainly performed by targeting the proximity between VDAC1 and IP3R1, and we confirmed some results by targeting VAPB-PTPIP51, another protein tether of MAMs[3]. Indeed, VDAC is located at the outer mitochondrial membrane and IP3R at the ER membrane, and both proteins belong to the calcium channel mediating calcium transfer from the ER to mitochondria. Therefore, their proximity is a direct reflect of organelle interaction. Whereas both proteins have 3 isoforms, the isoform 1 of each protein is targeted as they are expressed in STC-1 cells. In STC-1 cells and ileal Glu-Venus organoids, assays were conducted using a red, fluorescent *in situ* PLA DUOLINK kit (Merck, Darmstadt, Germany). In tissues, the assays were carried in 4 µm paraffin sections of intestinal samples using a brightfield *in situ* PLA DUOLINK kit (Merck). In organoids and C57Bl/6J mice colons, PLA was followed by immunofluorescence targeted against GLP-1 (ab22625, Abcam, UK) to identify L cells. Antibodies for VDAC1 (Abcam, ab14734, mouse source), IP3R1 (Merck, 07-1210, rabbit source), VAPB (Proteintech, 66191, mouse source) and PTPIP51 (Proteintech, 20641, rabbit source) were validated by immunofluorescence and diluted in the dilution buffer from the Duolink kit. The dilutions of antibodies were depended on cell type and Duolink kit batch. Cellular PLAs were captured with a Zeiss inverted fluorescent microscope using the AxioVision program and quantified by the BlobFinder software (Centre for Image Analysis, Uppsala University). Organoids and tissue slides were photographed using a Leica Thunder microscope using LAS Software and were manually quantified using FiJi software to specifically assess contacts in fluorescent L cells. Masking was not carried out. All results are expressed as the number of dots per nucleus, folded against control. There are several points to consider regarding PLA experiments. Firstly, PLA experiments work better in fixed cells than in fixed tissues, and we always obtained a greater number of PLA dots in cells than in tissues, whatever was the tissue; therefore, we adapted the dilution of primary antibody. Secondly, PLA are semi-quantitative experiments (only experimental conditions in a same PLA experiment can be compared), and the efficacity of kits varies from batch to batch; therefore, we cannot compare PLA dots from an experiment to another one. Lastly, as tissues are more auto-fluorescent than cells, we used a brightfield PLA kit for tissue, whereas we used a fluorescent PLA kit for cells;

therefore, we did not use the same microscope for imaging PLA in cells and tissues. For all these reasons, PLA images cannot be compared between figures.

Transmission electronic microscopy (TEM)

After treatments, STC-1 cells were fixed for 15 min in cold 4% glutaraldehyde, and then stored in a 1:1 mix of 4% glutaraldehyde/0.2M cacodylate at 4°C. Then, samples were post-fixed in 1% osmium tetroxide for 1h at 4°C, dehydrated and embedded in Epon. The samples were then cut using an RMC/MTX ultramicrotome (Elexience), and ultrathin sections (60-80 nm) were mounted on copper grids, contrasted with 8% uranyl acetate and lead citrate, and observed with a MegaView II high-resolution transmission electron microscopy camera. 15 pictures were taken at 40.000x magnification for each of the three triplicates. Masking was not carried out. Endoplasmic Reticulum (ER) and mitochondria were delimited using the FiJi software and the fraction of mitochondrial membrane in contact closer than 50nm with ER was normalized to the mitochondrial perimeter and expressed as % of mitochondrial membrane. We also gated the contacts to assess 20-30nm contacts that are better associated with calcium transfer. Results are the mean of all analysed mitochondria.

Calcium imaging

Reticular and mitochondrial calcium accumulation was measured 36 h after the expression of the D4ER or 4mt-D3CPV probes (FRET-based ratiometric calcium probes), respectively, using adenovirus (Ad-D4ER or Ad-4mtD3CPV) in a saline buffer in the presence or absence of extracellular calcium (2mM). After 2-min basal fluorescence measurement, either glucose (5mM), DCA (30µM), KCl (30mM, to induce membrane depolarization), or acetylcholine (ACh, 100µM, to stimulate ER-mitochondria calcium exchange) was added in puff, and acquisitions were continued during 2 min on 1 field/dish. Thapsigargin (2µM) was used to deplete ER calcium store and MCUi11 (20µM) to inhibit mitochondrial calcium entrance through the MCU. The fluorescence ratio “yellow fluorescent protein/cyan fluorescent protein” (YFP/CFP) was analysed with MetaFluor 6.3 (Universal Imaging) after removing background fluorescence. Masking was not carried out. Results represent the average of all analysed cells in 1-3 dishes/condition from 2-6 independent experiments.

Mitochondrial oxygen consumption

Oxygen consumption rate (OCR, pmoles/min) was measured using a Seahorse XFe 24 metabolic flux analyser (Agilent Technologies) according to manufacturer’s protocol. Cells

were plated in a Seahorse 24-well cell culture microplate at a density of 5.10^4 cells per well and pretreated with different compounds before the seahorse assays in HEPES medium. Cells were then washed and equilibrated in HEPES medium supplemented with 2mM glutamine, 2mM sodium pyruvate and 10mM glucose (Agilent Technologies) at 37°c in a CO_2 free incubator for 1h prior to a seahorse assay. Mitochondrial respiration measurements were performed in supplemented HEPES medium using the following concentration of sequentially injected compounds: mitochondrial ATP synthase inhibitor oligomycin 1.5 µM, mitochondrial uncoupler FCCP 1µM and a mix of mitochondrial respiratory chain inhibitors rotenone/antimycin A (1 µM each). The OCR was monitored over time under basal conditions and after addition of each compound, with 3 measurements of OCR for a period of 3 min. Measurements were normalized by a direct imaging of the cells via Hoescht-stained nuclei using Cytation 1 Cell Imaging Reader (BioTek). Data were analysed using Agilent Seahorse Wave version 2.6 and Agilent Seahorse Imaging and Cell Counting softwares. Masking was not carried out.

Mouse experiments

The mice were maintained at 25 °C and on an artificial 12h light–dark cycle, with the light period beginning at 7:30 am. Glu-Venus mice[4] were housed in with their littermates on sawdust bedding in plastic cages in an enriched environment. For the nutritional protocol, 5-week-old male C57Bl/6JOlaHsd mice (Envigo, France, 057) were separated in three groups (SD, HFHSD, HFHSD+Rosi), and fed the indicated diet (SD, 35.6% carbohydrates, 10.2% fat by mass, Genobios, France or HFHSD, 260-HF from SAFE, 33.2% carbohydrate, 39.4% fat, 18.8 % protein, in mass) for 16 weeks. The latter group was also given rosiglitazone by daily gavage (10mg/kg) during the last 6 weeks of the protocol. No randomisation was carried out. No animal was excluded, except for the insulin tolerance test (ITT) when low blood glucose levels during the test forced us to interrupt the test. During the sixteenth week, mice were submitted to both an intraperitoneal glucose and an insulin tolerance test (GTT and ITT, respectively), 3 days apart from each other. They were fasted overnight prior to being anesthetized with isoflurane and euthanized by cervical dislocation, and tissues of interest were removed and fixed. In addition, Glu-Venus mice were used for 2 independent protocols. For the acute treatment with glucose, 11 to 15-weeks-old female Glu-Venus mice were 6-h fasted, then force-fed with either 0,9% NaCl or 2mg/g glucose or 15mg/kg DCA, anesthetized with buprenorphine and ketamine/xylazine, and euthanized 30 min after the force-feeding. Blood was drawn from the portal vein and directly put in contact with 1% DPP4 inhibitor and EDTA.

Ileum and colon were collected, fixed in 4% PFA and embedded in paraffin. For the nutritional protocol in Glu-Venus mice, 5-week-old male Glu-Venus mice were fed with either a SD of HFHSD for 16 weeks. During the sixteenth week, mice were submitted to both a GTT and an ITT, at least 3 days apart from each other. The week after, mice were fasted for 6 h, then force-fed with either 0,9% NaCl or 2mg/g glucose, anesthetized with buprenorphine and ketamine/xylazine, and euthanized 30 min after the force-feeding. Blood was drawn from the portal vein and directly put in contact with 1% DPP4 inhibitor and EDTA. Ileum and colon were collected, fixed in 4% PFA and embedded in paraffin.

Statistical analyses

Statistical analyses were performed using GraphPad 8. Comparison between 2 groups were analysed using unpaired T-test or Mann-Whitney. Comparisons between more than 2 groups were analysed using ANOVA followed by Sidak's multiple comparisons' test if the data follow a normal distribution; otherwise, the data were analysed using the Kruskal-Wallis test followed by Dunn's multiple comparison test. Statistical tests used in all analysis were two-sided. Outliers were removed based on ROUT' test. In vitro experiments were performed in 2-3 independent experiments (=N) in several replicate (n) unless stated otherwise. For single cell imaging analysis, statistics were performed on n=number of cells to assess single cell effect as well as heterogeneity between them. Please note that for figures where we combined results from different treatments, either performed independently or within the same experiment, we expressed the results as a fold change and depicted the control condition as a dotted line. For figures where all conditions were performed during the same experiment, the control histogram is shown. Both N and n values, as well as the statistical tests, are indicated in the figure legends. Significance was defined as a value of $p<0.05$.

Abbreviations

2-APB: 2-aminoethyldiphenylborate; ACh: Acetylcholine; CFP: cyan fluorescent protein; DCA: Deoxycholic acid; DMSO: dimethyl sulfoxide; DPP4: Dipeptidyl-peptidase-4; ELISA: Enzyme-Linked Immunosorbent Assay; ER: Endoplasmic Reticulum; Ex4: exendin 4; FATE1: Fetal and Adult Testis Expressed 1; GLP-1: Glucagon-like peptide-1; HFHSD: High Fat High Sucrose Diet; MCU: mitochondrial uniporter; αMG: methyl-α-D-glucopyranoside; OCR: Oxygen consumption rate; PFA: Paraformaldehyde; PLA: Proximity Ligation Assay; Rosi:

Rosiglitazone; SD: Standard Diet; TEM: Transmission Electronic Microscopy, YFP: yellow fluorescent protein.

References

1. Beaulant, A. et al. Endoplasmic reticulum-mitochondria miscommunication is an early and causal trigger of hepatic insulin resistance and steatosis. J. Hepatol. 77, 710–722 (2022)
2. Doghman-Bouguerra M. et al. FATE1 antagonizes calcium- and drug-induced apoptosis by uncoupling ER and mitochondria. EMBO Rep.17(9):1264-80 (2016).
3. De Vos KJ, Mórotz GM, Stoica R, Tudor EL, Lau KF, Ackerley S, Warley A, Shaw CE, Miller CC. VAPB interacts with the mitochondrial protein PTPIP51 to regulate calcium homeostasis. Hum Mol Genet. 15;21(6):1299-311 (2012).
4. Reimann, F. et al. Glucose Sensing in L Cells: A Primary Cell Study. Cell Metab. 8, 532–539 (2008).

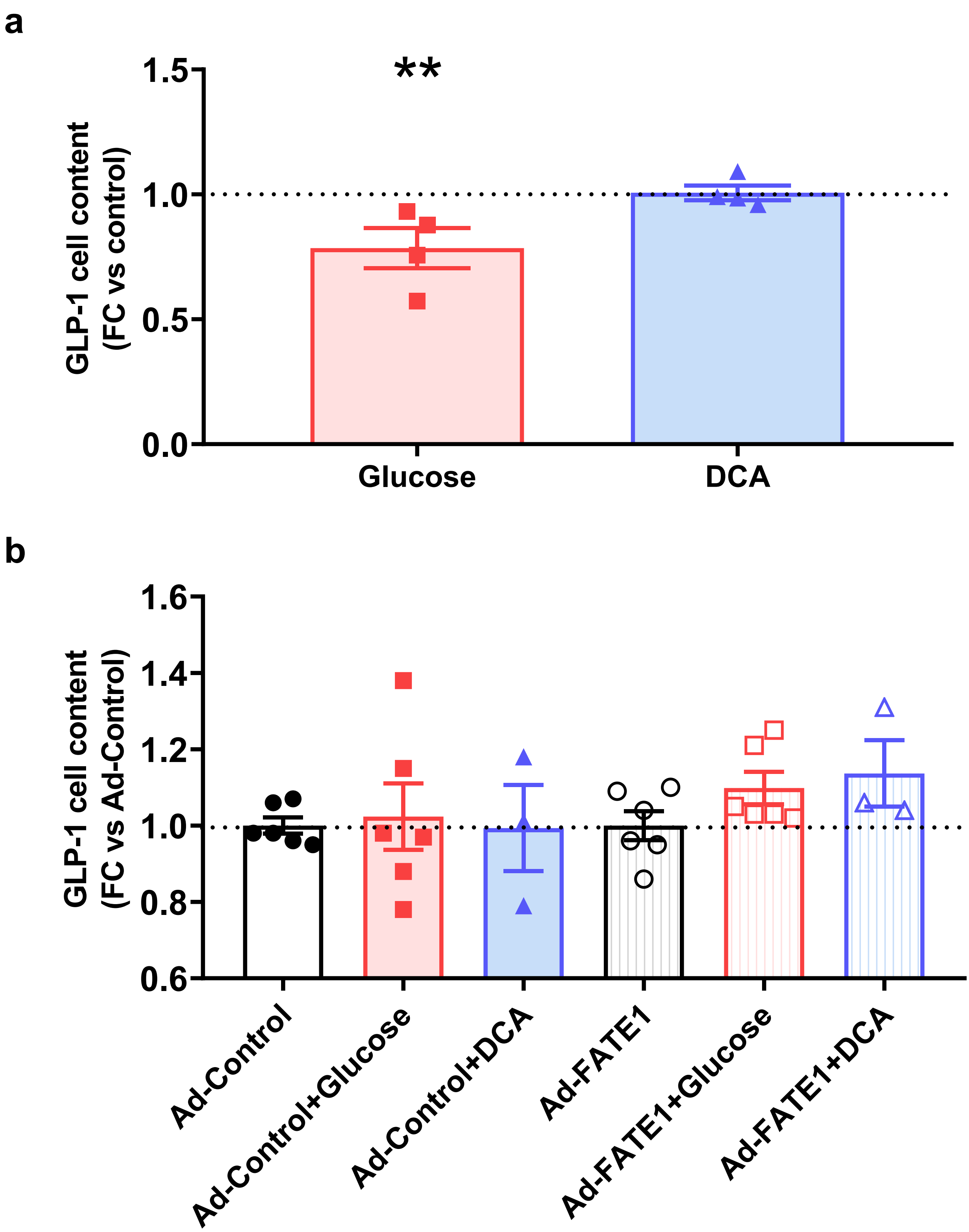

ESM Figure 1: GLP-1 content after acute (a) or chronic (b) modulation of MAMs. Active GLP-1 was quantified by ELISA into STC-1 cells following acute modulation of MAMs by GLP-1 secretagogues (a) or chronic modulation of MAMs by FATE1 expression (b). Secreted GLP-1 secretions in the same conditions were illustrated in Figure 1a and 2h, respectively. Data are expressed as fold change (FC) vs respective control, N= 4 for (a) and n=3-6 in N=1-2 experiment(s) for (b). ** p<0.01 (Kruskal-Wallis test). Ad, adenovirus; DCA, Deoxycholic Acid; FATE1, fetal and adult testis expressed 1; FC, fold change.

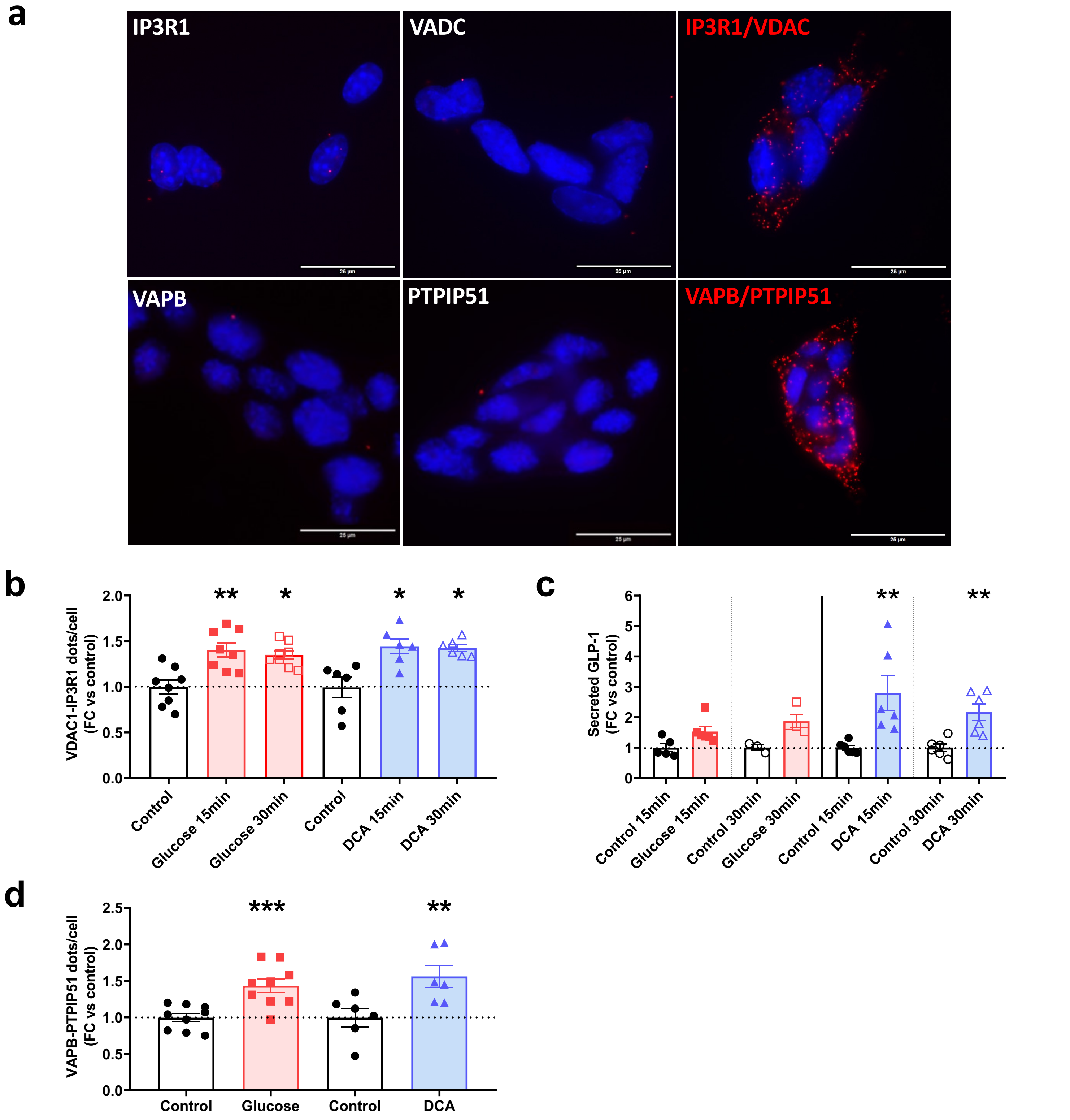

ESM Figure 2: Additive experiments to validate of the use of *in situ* PLA to analyse ER-mitochondria interactions and analysis of the dynamic effect of glucose and DCA on MAMs. a) Illustrative images validating that no PLA dots were apparent in STC-1 cells when only one antibody was used, whereas red-fluorescent dots were present when both antibodies were present, scale bar at 25 µm. b-c) Acute glucose and DCA effects on MAMs (b), measured by *in situ* PLA, and on GLP-1 secretion (c), measured by ELISA, in STC-1 cells. Data are expressed as fold change (FC) vs respective control, n=3-8 in N=1-3 experiment(s). d) Effect of glucose and DCA on MAMs by targeting by *in situ* PLA the VAPB-PTPIP51 tether. Data are expressed as FC vs respective control, n=6-9 in N=2-3 experiments. * $p<0.05$, ** $p<0.01$ and *** $p<0.001$ (Kruskal-Wallis test followed by Dunn's post-test for (b) and Mann-Whitney test for (c & d)). DCA, Deoxycholic Acid; FC, fold change.

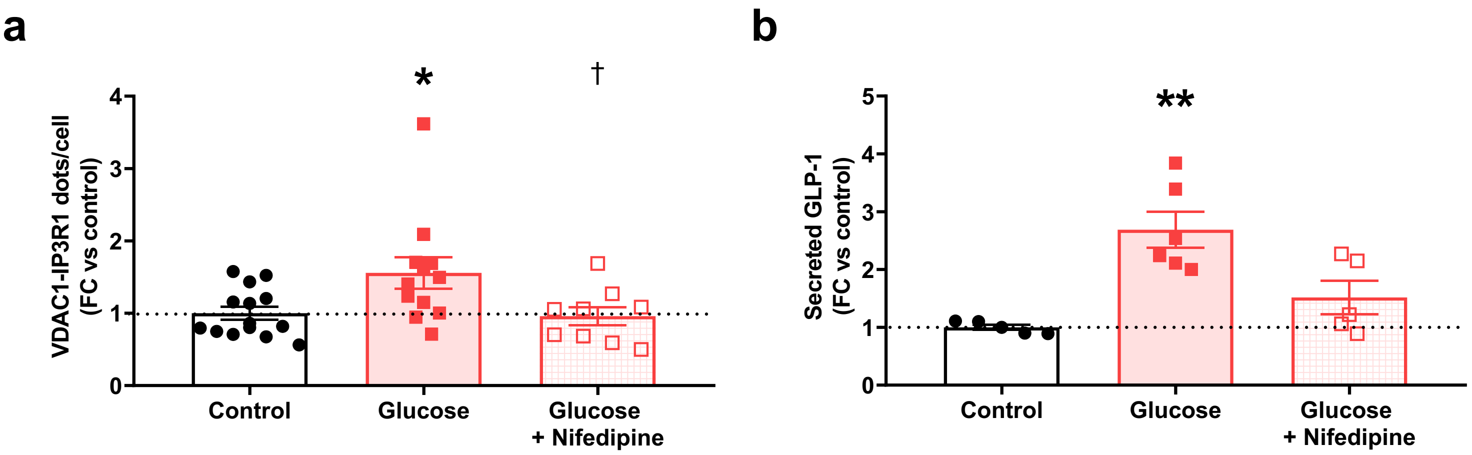

ESM Figure 3: Effect of glucose on MAMs and GLP-1 secretion in GLUTag cells. GLUTag cells were incubated for 1h with glucose in absence or presence of nifedipine (5µmol/l) and the effects on MAMs (a), by targeting VDAC1-IP3R1 proximity, and on GLP-1 secretion (b) were analysed. Nifedipine treatment prevented the effect of glucose on MAMs and GLP-1 release, validating the signalling pathway identified in STC-1 cells. Data are expressed in fold change (FC) vs control. a) n=9-14 in N=3-4 experiments; b) n=5-6 in N=3 experiments. * p<0.05, ** p<0.01 for glucose effect vs control; † p<0.05 for nifedipine effect vs glucose (Kruskal-Wallis test followed by Dunn's post-test). FC, fold change.

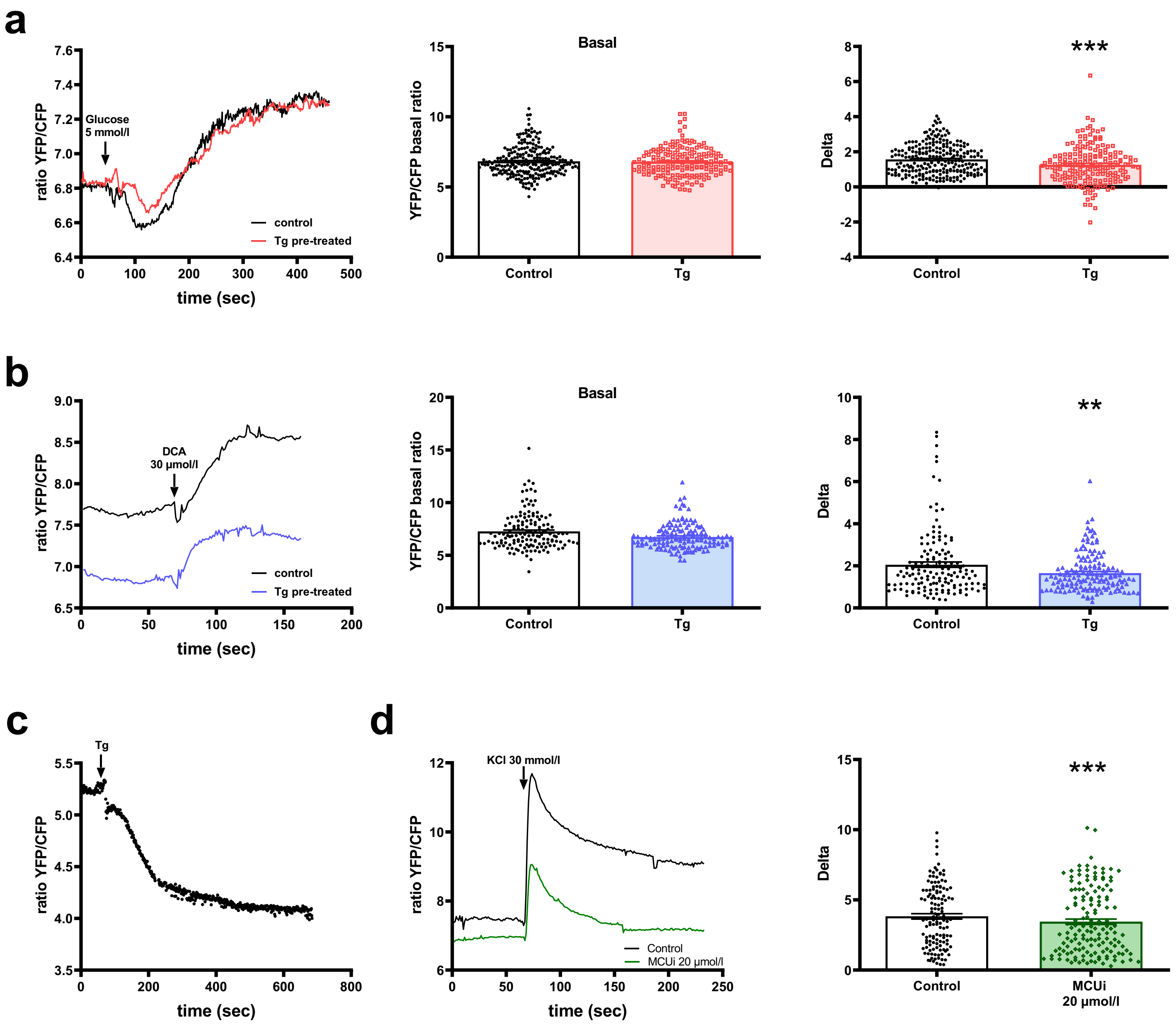

ESM Figure 4: Complementary analyses on calcium measurements in STC-1 cells. STC-1 cells were infected with Ad-4mtD3CPV (mitochondria-specific calcium probe) or Ad-D4ER (reticular calcium probe) for 36h, before treatments. All calcium measurements were performed in presence of extracellular calcium (2 mmol/l). a-b) Acute effect of glucose 5 mmol/l (a) or DCA 30 µmol/l (b) on mitochondrial calcium levels, measured in absence or presence of thapsigargin (Tg, 2 µmol/l, added 10 min before). Calcium was analysed in 182 cells for (a) and 140 cells for (b). c) Ad-D4ER-infected STC-1 cells were acutely stimulated with Tg (2 µmol/l) and ER calcium was monitored (representative experiment). Ten minutes of treatment are required to deplete ER calcium levels. d) Ad4mtD3CPV-infected STC-1 cells were pre-treated or not with MCUi11 20 µmol/l (specific inhibitor of MCU) and then stimulated with KCl 30 mmol/l to induce membrane depolarization. Calcium was analysed in 79 to 182 cells. Data +/- SEM, N=2-4 experiments. ** p<0.01, *** p<0.001 vs respective control (unpaired T-test). CFP, cyan fluorescent protein; DCA, Deoxycholic Acid; Tg, thapsigargin; YFP, Yellow fluorescent protein.

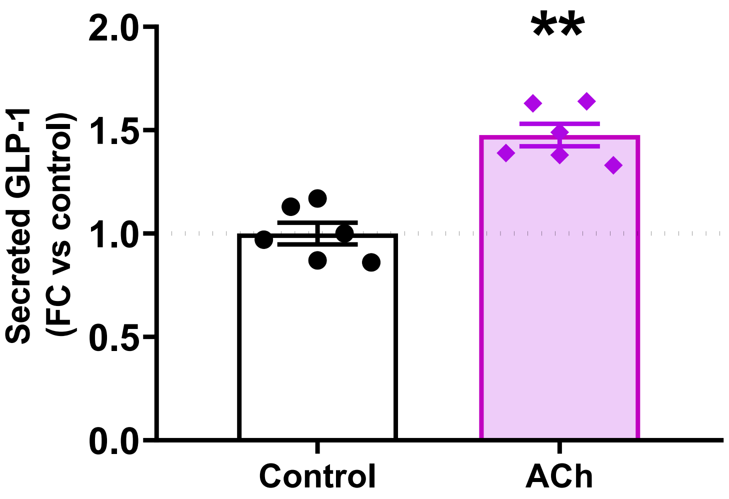

ESM Figure 5: Effect of acetylcholine treatment on GLP-1 secretion in STC-1 cells. STC-1 cells were incubated with acetylcholine (ACh, 100 µmol/l) for 1 hour, and GLP-1 secretion was measured by ELISA. Data are expressed as fold change (FC) vs control, n= 6 in N=2 experiments. ** p<0.01 (Mann-Withney test). Ach, acetylcholine; FC, fold change.

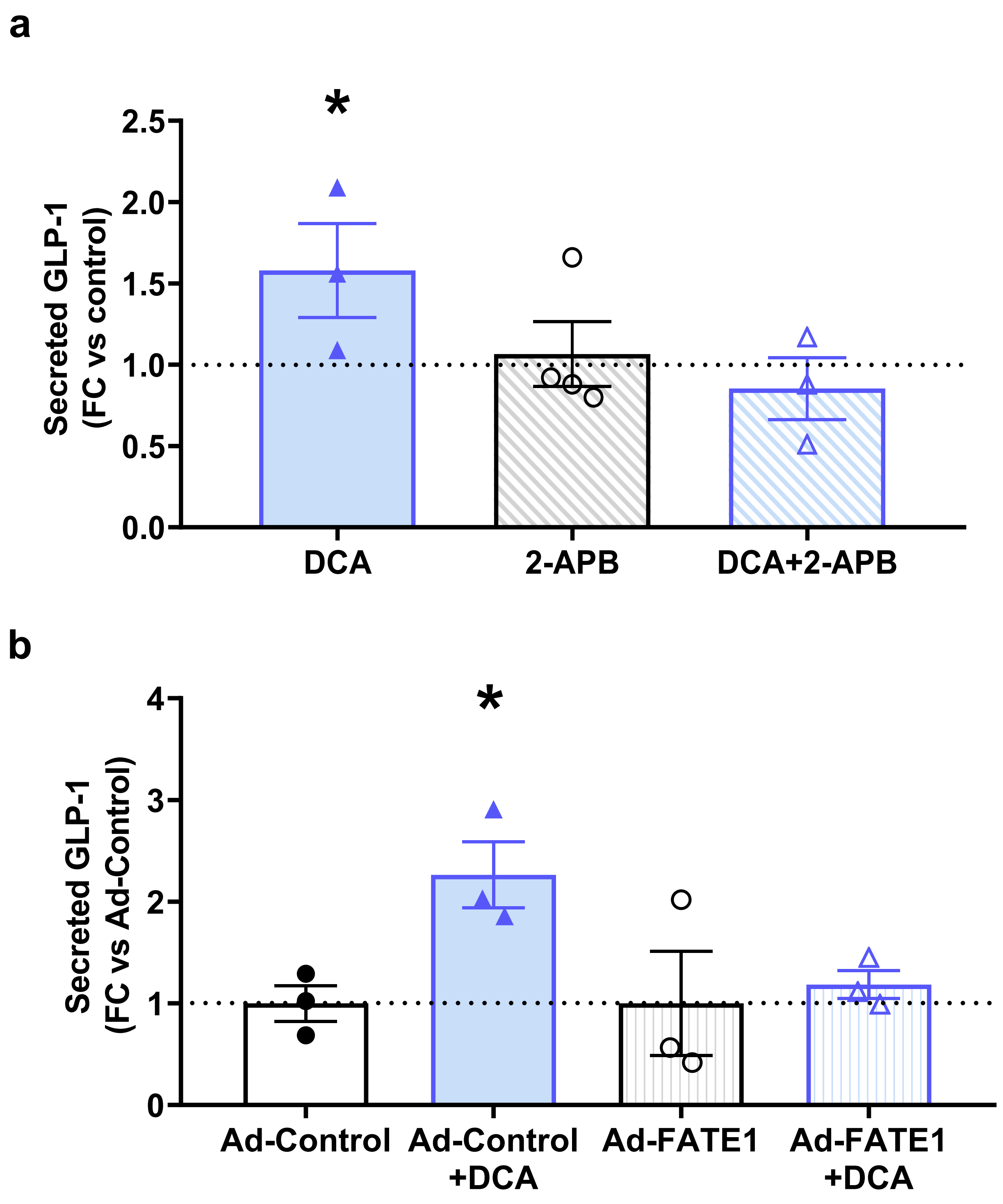

ESM Figure 6: Role of MAMs in DCA-induced GLP-1 secretion. a) STC-1 cells were treated with DCA (30 µmol/l) for 1h in absence or presence of 2-APB (50 µmol/l, inhibitor of IP3R), and GLP-1 secretion was quantified by ELISA. b) STC-1 cells were infected with Ad-control or Ad-FATE1 for 48h and then stimulated with DCA for 1h, and GLP-1 secretion was quantified by ELISA. Data are expressed as fold change (FC) vs respective control, N=3-4 for (a) and n=3 in N=1 experiment for (b). * p<0.05 (Two-way ANOVA test followed by Sidak post-test). 2-APB, 2-aminoethyldiphenylborate; Ad, adenovirus; DCA, Deoxycholic Acid; FATE1, fetal and adult testis expressed 1; FC, fold change.

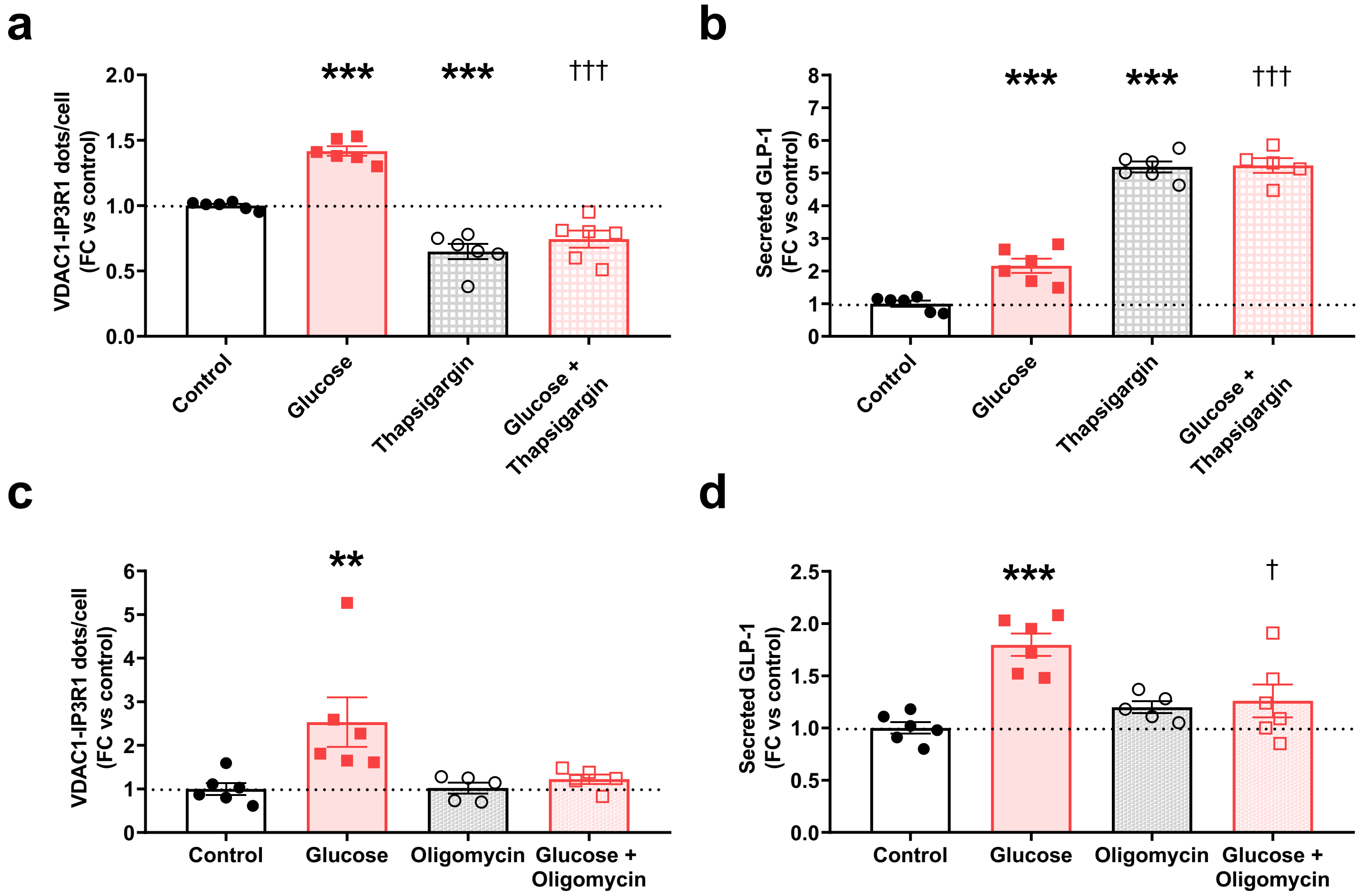

ESM Figure 7 : Repercussion of ER stress and mitochondrial dysfunction on glucose-induced GLP-1 secretion. a-b) STC-1 cells were pre-treated with thapsigargin (0,3 µmol/l) for 6h to induce ER stress, and then stimulated with or without glucose for 1h. Then, MAMs were quantified by in situ PLA (a) and GLP-1 secretion by ELISA (b). c-d) STC-1 cells were pre-treated with oligomycin (0,5 µmol/l) for 1h to induce mitochondrial stress, and then stimulated with or without glucose for 1h. Then, MAMs were quantified by *in situ* PLA (c) and GLP-1 secretion by ELISA (d). Data are expressed as fold change (FC) vs respective control, n=5-6 in N=2 experiments. ** $p<0.01$ and *** $p<0.001$ for treatment effect vs control; † $p<0.05$ and ††† $p<0.001$ for drug effect vs glucose (Two-way ANOVA followed by sidak post-test). FC, fold change.

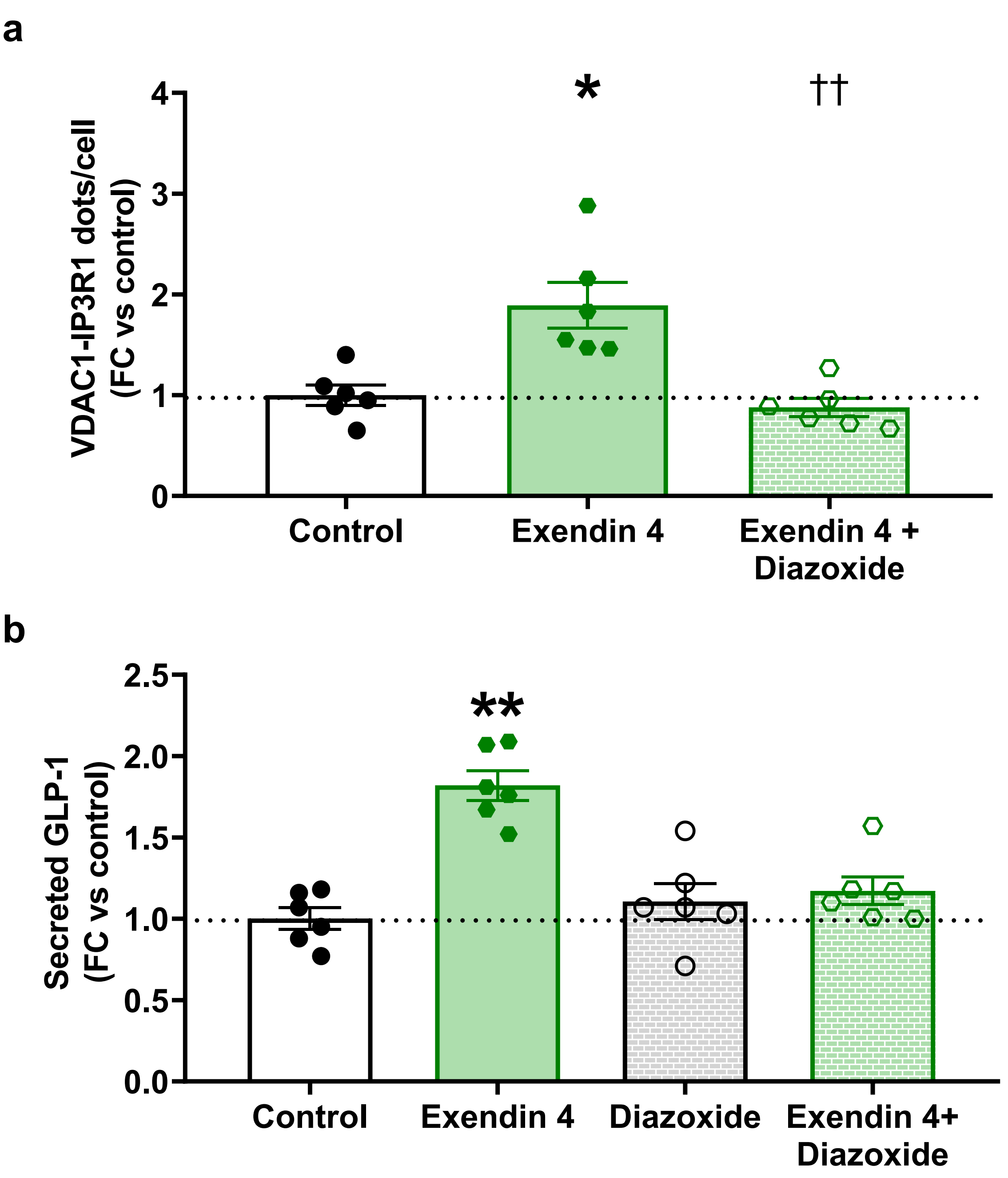

ESM Figure 8 : Link between the GLP-1R and the electrogenic pathway in STC-1 cells. a-b) STC-1 cells were treated with exendin 4 (Ex4, 100 nmol/l, GLP-1R agonist) for 1h in the absence or presence of diazoxide (100 µmol/l, forces the opening of K_{ATP} channel), and then MAMs were measured by *in situ* PLA and GLP-1 secretion by ELISA. Data are expressed as fold change (FC) vs respective control, n=6 in N=2 experiments. * $p<0.05$ and ** $p<0.01$ for Ex4 effect vs control; †† $p<0.01$ for diazoxide effect vs Ex4 (Kruskal-Wallis test followed by Dunn's post-test). FC, fold change.

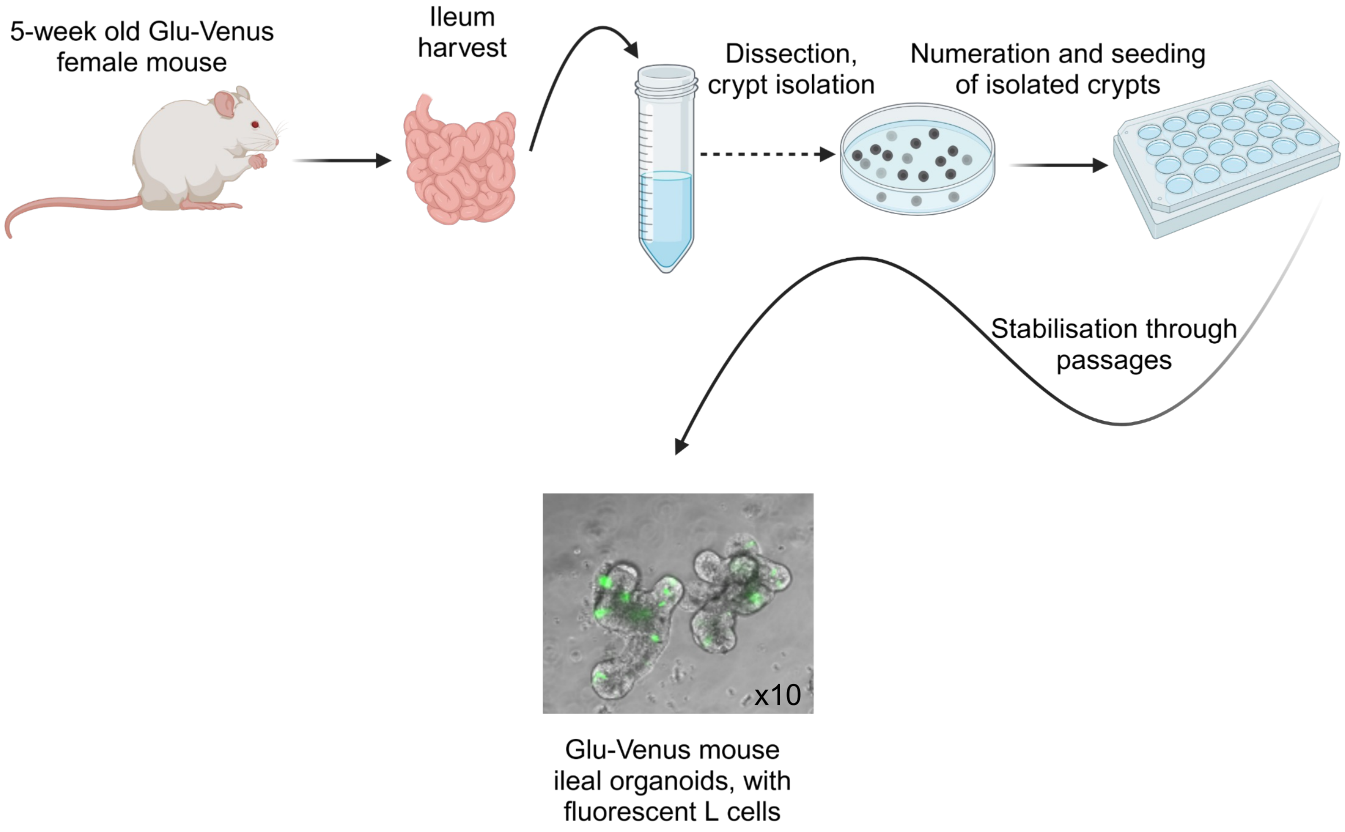

ESM Figure 9: Schematic representation of the methods used to develop ileal organoids from Glu-Venus mice, and illustration of Venus fluorescence. Created with BioRender.com.

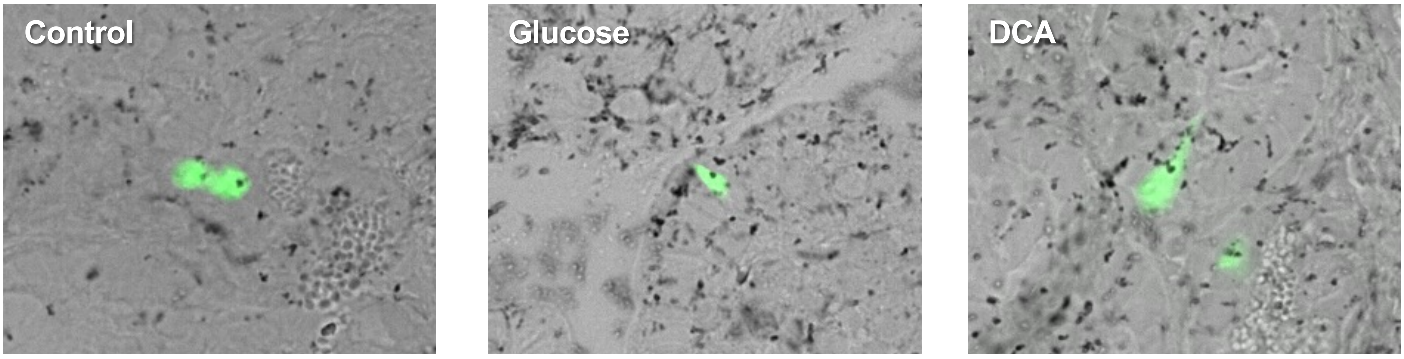

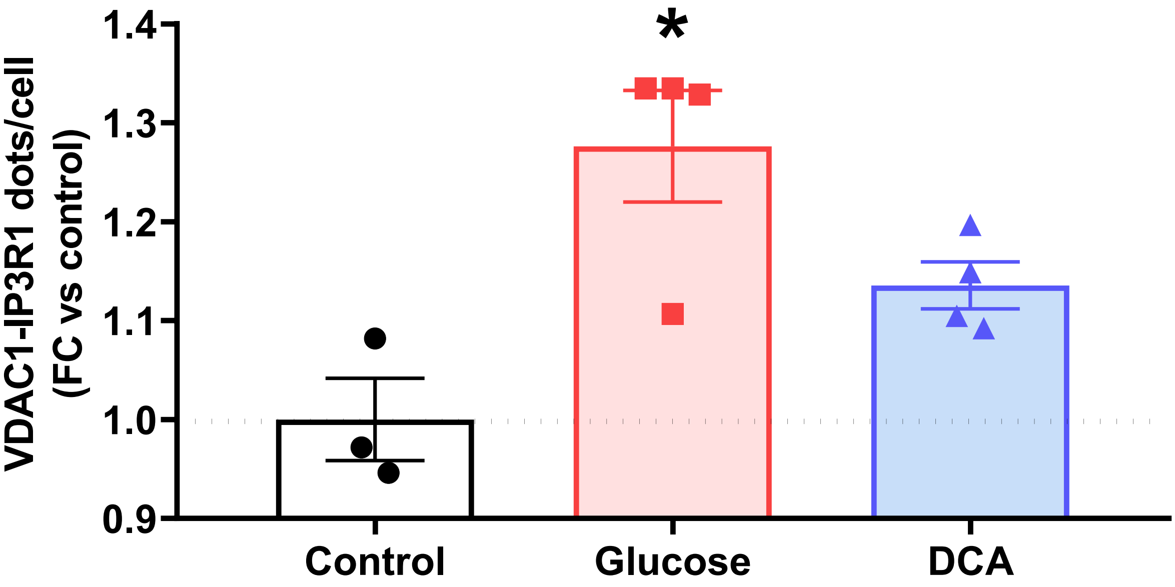

ESM Figure 10: The induction of MAMs after an oral gavage is confirmed in ileal L cells in healthy female Glu-Venus mice. ER-mitochondria interaction quantified by in situ PLA in fluorescent ileal L cells 30 minutes after an oral gavage of Glu-Venus female mice. Illustrative images of PLA experiment in ileal L cells from control, glucose- and DCA-treated mice (Venus-positive L cells are indicated in green; VDAC1-IP3R1 dots appear in black) and quantification of VDAC1-IP3R1 dots/cell in Venus-positive L cells. N=3-4 mice. * $p<0.05$ (Kruskal-Wallis test followed by Dunn's multiple comparisons). DCA, Deoxycholic Acid; FC, fold change.

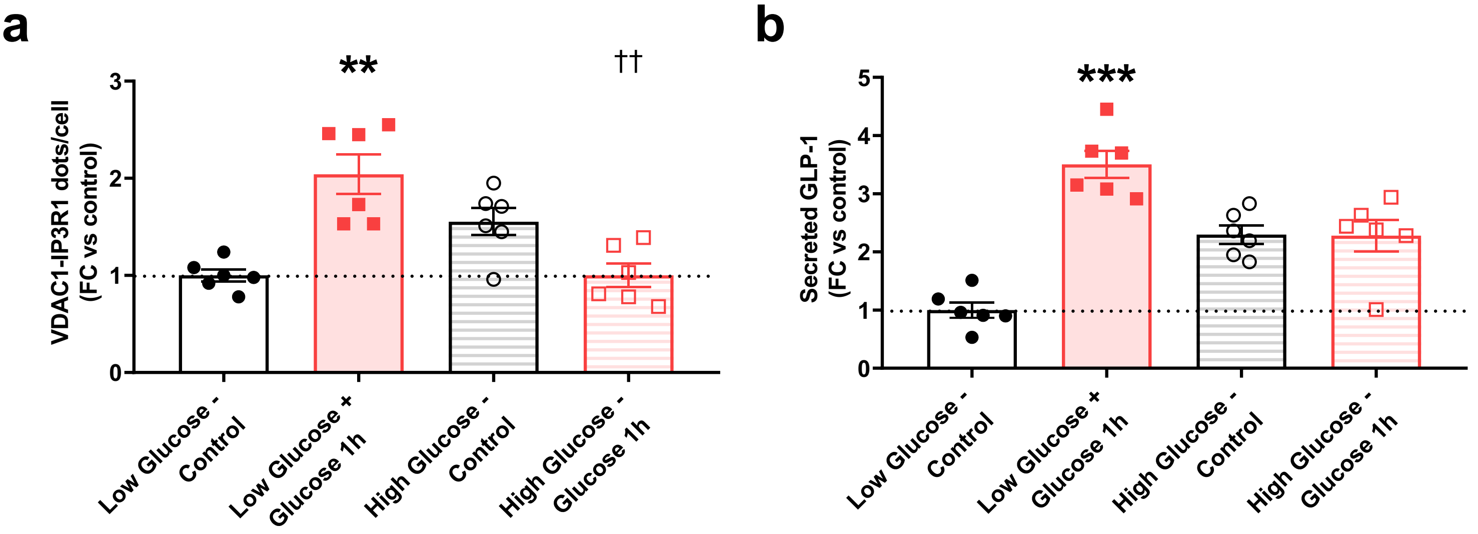

ESM Figure 11: Effect of chronic high glucose treatment on MAMs and GLP-1 secretion in STC-1 cells. STC-1 cells were incubated or not with high glucose (HG) concentration (4,5 g/l) for 48 hours and then further stimulated with glucose in HEPES buffer for 1 hour. Effect of HG treatment on glucose-mediated regulation of MAMs (a) and GLP-1 secretion (b). Chronic glucotoxicity concomitantly prevents the dynamic regulation of MAMs and GLP-1 secretion by glucose, confirming data observed in HFHSD mice. Data are expressed as fold change (FC) vs control, n=6 in N=2 experiments. ** $p<0.01$ and *** $p<0.001$ for treatment effect vs respective control; †† $p<0.01$ for high glucose effect vs low glucose effect (Kruskal-Wallis test followed by Dunn's post-test). FC, fold change.
